# Direct Observation of Contact Reaction Induced Ion Migration and its Effect on Non‐Ideal Charge Transport in Lead Triiodide Perovskite Field‐Effect Transistors

**DOI:** 10.1002/smll.202302494

**Published:** 2023-06-10

**Authors:** Youcheng Zhang, Amita Ummadisingu, Ravichandran Shivanna, Dionisius Hardjo Lukito Tjhe, Hio‐Ieng Un, Mingfei Xiao, Richard H. Friend, Satyaprasad P. Senanayak, Henning Sirringhaus

**Affiliations:** ^1^ Optoelectronics Group Cavendish Laboratory University of Cambridge J.J. Thomson Avenue Cambridge CB3 0HE UK; ^2^ Cambridge Graphene Centre Department of Engineering University of Cambridge 9 JJ Thomson Ave Cambridge CB3 0FA UK; ^3^ Department of Physics Indian Institute of Technology Madras Chennai 600036 India; ^4^ Nanoelectronics and Device Physics Lab School of Physical Sciences National Institute of Science Education and Research An OCC of HBNI Jatni 752050 India

**Keywords:** contact reaction, ion migration, non‐ideal charge transport, perovskites field‐effect transistors (FETs), photoluminescence and EDX mapping

## Abstract

The migration of ionic defects and electrochemical reactions with metal electrodes remains one of the most important research challenges for organometal halide perovskite optoelectronic devices. There is still a lack of understanding of how the formation of mobile ionic defects impact charge carrier transport and operational device stability, particularly in perovskite field‐effect transistors (FETs), which tend to exhibit anomalous device characteristics. Here, the evolution of the *n*‐type FET characteristics of one of the most widely studied materials, Cs_0.05_FA_0.17_MA_0.78_PbI_3,_ is investigated during repeated measurement cycles as a function of different metal source–drain contacts and precursor stoichiometry. The channel current increases for high work function metals and decreases for low work function metals when multiple cycles of transfer characteristics are measured. The cycling behavior is also sensitive to the precursor stoichiometry. These metal/stoichiometry‐dependent device non‐idealities are correlated with the quenching of photoluminescence near the positively biased electrode. Based on elemental analysis using electron microscopy the observations can be understood by an *n*‐type doping effect of metallic ions that are created by an electrochemical interaction at the metal–semiconductor interface and migrate into the channel. The findings improve the understanding of ion migration, contact reactions, and the origin of non‐idealities in lead triiodide perovskite FETs.

## Graphical abstract

Organic–inorganic lead triiodide perovskites often exhibit non‐ideal charge transport properties. When under electrical bias, the current of a perovskite field‐effect transistor can increase or decrease. This study reveals that these properties originate from electrode reaction induced ion migration and doping processes at electrode interface and are strongly affected by sample stoichiometry and metal contact work function.

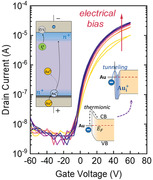

## 1 Introduction

Eliminating defects has been a major issue in improving the performance and operational stability of metal halide perovskite‐based optoelectronic devices. Elimination of interfacial^[^ ^1^ ^]^ and lattice defects^[^ ^2^ ^]^ in perovskite solar cells alongside careful compositional engineering has resulted in the achievement of power conversion efficiencies exceeding 25%. Methylammonium lead triiodide (MAPbI_3_) is known to have mobile point defects, such as charged vacancies and interstitials of the methylammonium cation ($V_{\mathrm{MA}}^{-}$, ${\mathrm{MA}}_{i}^{+}$) and iodine anion ($V_{I}^{+}$, $I_{i}^{-}$), which can cause device instabilities and act as non‐radiative recombination centers.^[^ ^3^, ^4^ ^]^ The interaction between MAPbI_3_ and metal surfaces can lead to complex ion migration and electrochemical processes that can lead to device operational instabilities. When MAPbI_3_ is in direct contact with metal surfaces, it can react with the metal to produce various products such as Pb^0^, CH_3_NH_2_, I_2_,^[^ ^5^ ^]^ and metal ions (e.g., Au^+^).^[^ ^6^, ^7^, ^8^, ^9^, ^10^ ^]^ These reactions can occur under the influence of an electrical field and can contribute to the transformation of ions in MAPbI_3_ into redox and decomposition products such as $I_{i}^{0}$, $I_{i}^{+}$,^[^ ^11^, ^12^ ^]^ CH_3_NH_2_,^[^ ^7^ ^]^ CH_3_^+^ and NH_3_^+^.^[^ ^13^ ^]^ The complexity of these ion migration and electrochemical processes presents a challenge to the operational stability in perovskite‐based optoelectronic devices.

Field‐effect transistors (FETs) are a suitable lateral geometry platform for the characterization of long‐range transport of charge carriers and are sensitive to both ionic defects and contact electrochemical reactions. Performances of early reported MAPbI_3_ FETs were severely affected by ion migration, with room temperature mobility values below 10^−4^ cm^2^ V^−1^ s^−1^ and large hysteresis in the transfer curve.^[^ ^14^, ^15^ ^]^ Later work improved device performance and realized FET mobility values >1 cm^2^ V^−1^ s^−1[^ ^16^, ^17^, ^18^ ^]^ by implementing strategies such as replacing methylammonium cation (MA^+^) with formamidinium (FA^+^) and cesium cations (Cs^+^),^[^ ^16^, ^19^, ^20^ ^]^ grain‐stabilizing additives,^[^ ^18^, ^21^ ^]^ surface cleaning,^[^ ^16^, ^17^ ^]^ as well as the engineering of dielectrics^[^ ^18^, ^21^, ^22^ ^]^ and metal contacts.^[^ ^17^, ^23^ ^]^ Despite the remarkable increase in FET mobility values, the bias stress stability remained relatively low with large threshold voltage shifts |Δ*V*_th_| ≥ 5 V.^[^ ^16^, ^17^ ^]^ Improving the operational stability of lead triiodide‐based perovskites requires a thorough understanding of the defect dynamics under bias in lateral devices. Additionally, perovskite FETs are often evaluated under conditions that reduce these instabilities, such as rapid sweep rates or pulsed measurements.^[^ ^24^ ^]^ Bias‐stress measurements can provide valuable insights into the interaction between mobile ions and electronic charges and reveal transport behaviors that are not commonly observed in other semiconductors. For example, Canicoba et al. observed a thousand‐fold increase of ON‐OFF ratio on MAPbI_3_ FETs after gate bias and attributed it to the doping effect of mobile ions.^[^ ^22^ ^]^

In this study, we examine the development of anomalous charge transport behavior in mixed‐cation lead triiodide perovskite FETs in a bottom‐gate, bottom‐contact geometry (BGBC) when subjected to multiple measurement cycles. We find that the operational stability of these FETs is greatly influenced by the choice of source–drain metal electrodes and the solution precursor stoichiometry. By tuning the solution stoichiometry, we can systematically alter the chemical environment and defect composition of the films and study the ensuing changes in charge transport parameters, photoluminescence signal, and morphology at perovskite‐contact interfaces with different metal electrodes during electrical bias. We use ex situ elemental analysis on the devices to gain a thorough understanding of electrode degradation and the migration of metallic ions from the electrodes during operational instabilities. Finally, we propose a metallic ion‐induced *n*‐type doping mechanism to explain the observed unusual transport behaviors.

## 2 Results

**Figure 1:**
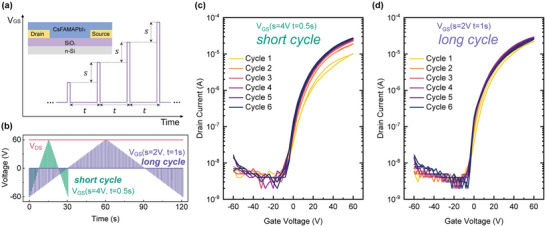
Pulsed‐mode transfer characteristics measurements for Cs_0.05_FA_0.17_MA_0.78_PbI_3_ (+5 mol.% extra PbI_2_). a) Schematic illustration for gate voltage changes in pulsed mode measurements with a certain voltage increment step *s* and pulse interval time *t*. The insert shows the cross‐section of the BGBC FET geometry; b) temporal variation of *V*_DS_ and *V*_GS_ in one measurement cycle with two combinations of (*s*,*t*). Transfer characteristics of Cs_0.05_FA_0.17_MA_0.78_PbI_3_ (with +5 mol.% excess PbI_2_) of six consecutive c) short cycles (*s* = 4 V, *t* = 0.5 s) and d) long cycles (*s* = 2 V, *t* = 1 s). Channel length *L* = 100 µm, channel width *W* = 1 mm. *V*_DS_ = +60 V, *V*_G_ sweeps from −60 to +60 V to −60 V.

We selected Cs_0.05_FA_0.17_MA_0.78_PbI_3_ as the material composition for this study. Although its mobility is not among the highest values reported for perovskite FETs, it is a widely studied system for optoelectronic devices, such as solar cells, and also provides relatively stable and reproducible *n*‐type FETs with moderately high mobilities on the order of 0.2 cm^2^ V^−1^ s^−1^ at room temperature.^[^ ^16^ ^]^ We first characterized the stability of the FET characteristics upon repeated cycling of the transfer characteristics by using a composition with 5% molar excess of lead iodide (PbI_2_) and gold source–drain electrodes. The gate‐voltage (*V*_GS_) was applied in square‐wave pulses (pulse width = 1 ms) with a pulse interval time *'t'* and voltage increment step *'s'*, as shown in **Figure** **1**a. The duration of each measurement cycle can be short (30 s) or long (120 s) depending on the choice of *s* and *t*, while a +60 V source–drain bias (*V*_DS_) is constantly applied (Figure 1b) during the cycle. Two condition sets, (*s* = 4 V, *t* = 0.5 s) and (*s* = 2 V, *t* = 1 s), are referred to as short and long measurement cycles, respectively. Under these measurement conditions we can tune the lateral diffusion of ions, while minimizing effects of the vertical redistribution of ions due to the applied gate voltage.

The evolution of the transfer characteristics upon six short and long cycles is illustrated in Figure 1c,d for two separate devices from the same chip fabricated with gold electrodes and a 5% molar excess of PbI_2_ in the precursor solution. The FETs exhibit the expected *n*‐type characteristics with a well‐defined turn‐on at *V_GS_* = −10 V, a high ON–OFF current ratio of 10^3^–10^4^ and a moderately high mobility on the order of 0.2 cm^2^ V^−1^ s^−1^. In the short cycle measurements, the maximum drain current (*I*_D‐max_) increases threefold from ≈10 µA in the first cycle to ≈30 µA after six repeated runs. In the long‐cycle measurements, *I*_D‐max_ begins at ≈20 µA in the first cycle and quickly stabilizes at ≈30 µA in the following cycle. As the duration of a long cycle is four times longer than a short cycle, the variations of *I*_D‐max_ could depend on the device's history of source–drain bias and/or gate pulse. We attribute the significant increase in channel current during the short‐cycle measurements mainly to the application of a constant source–drain bias, rather than gate pulses. This is evident when comparing measurements only with source‐drain biasing history (Figure S1, Supporting Information). If FETs are pre‐source‐drain biased for 3.5 min without gate voltage applied they already exhibit *I*_D‐max_ ≈30 µA in their first scans, which is comparable to the *I*_D‐max_ (≈30 µA) obtained on samples after repeated transfer sweeps. The application of a gate voltage through a common gate has negligible effect on the performance of other devices which are not subjected to a source–drain bias as shown in Figure S2 (Supporting Information). Henceforth, we refer to this source‐drain bias induced current increase effect as “current build‐up effect” in the following.

**Figure 2:**
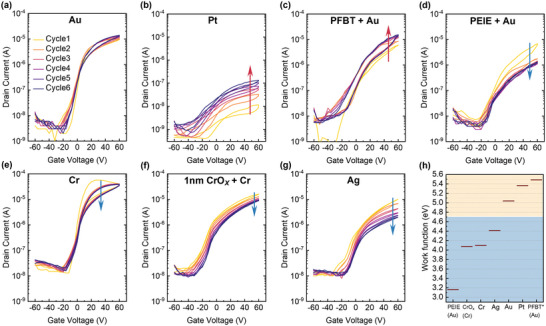
Transfer characteristics for Cs_0.05_FA_0.17_MA_0.78_PbI_3_ perovskites with different source‐drain electrodes. a) Gold. b) Platinum. c) PFBT modified gold. d) PEIE modified gold. e) Chromium. f) 1 nm CrO_x_ modified chromium. g) Silver. h) Work function values of the above used electron materials obtained from UPS measurement. Electrode materials used here are categorized into high work function materials (Au, Pt, PFBT‐Au) and low work function materials (PEIE‐Au, Cr, CrO_x_‐Cr, Ag). The ON current increases in high work function electrode devices and decreases in low work function devices. The work function value of PFBT‐Au is taken from Ref. [23].

We investigated the role of the source–drain contacts on the current build up effect and performed repeated short cycle measurements on devices by systematically varying the source–drain electrode material. This allowed us to access work functions ranging from 3.2 to 5.5 eV. The selection of the diverse electrodes was principally to cover a broad spectrum of work functions, while also considering the simplicity of their fabrication process and the compatibility with a reasonably high level of device performance (see the Experimental Section for technical detail). To minimize the contribution from excess ions, we used a stoichiometric composition compared to devices fabricated with slight excess of PbI_2_ as in Figure 1. **Figure** **2**a–g shows the evolution of transfer characteristics over six consecutive transfer sweeps for gold (Au), platinum (Pt), pentafluorothiophenol‐modified gold (PFBT‐Au), polyethylenimine ethoxylated‐modified gold (PEIE‐Au), chromium (Cr), 1 nm chromium oxide‐modified chromium (CrO_x_‐Cr), and silver (Ag). Figure 2h indicates the work functions of these electrodes measured by ultraviolet photoelectron spectroscopy (UPS). Figure S3 (Supporting Information) shows the UPS spectra of these electrodes.

In the stoichiometric sample, the drain current of gold electrode samples remains stable over six measurement cycles (Figure 2a), indicating good operational stability of these FETs, though we reproducibly observe clockwise hysteresis in the characteristics. Since Au has an intermediate work function value among the different electrode materials used to fabricate the FETs, we categorize the electrode materials as low or high work function materials by comparing them to the work function of Au. Interestingly, we observe that the devices fabricated with lower work function materials (PEIE‐Au, Cr, CrOx‐Cr, and Ag) show a decrease in current (up to 90%) while devices fabricated from high work function materials (Pt and PFBT‐Au) exhibit an increase in drain current upon multiple cycles of transfer sweeps. This trend is consistent with the fact that materials with low work functions tend to have high chemical reactivity at the metal–semiconductor (M–S) interface,^[^ ^25^, ^26^, ^27^, ^28^, ^29^, ^30^, ^31^, ^32^ ^]^ which may explain the drop in current in these materials due to reactions at the M–S interface. We also tested two additional types of metal electrodes: titanium (low work function) and gold‐palladium alloy (high work function). The work functions for these materials are shown in Figure S4 (Supporting Information). Figure S5 (Supporting Information) shows that the ON current decreases in Ti device and increases in Au/Pd device as we predicted. The extracted FET mobility for these devices also follows the same pattern as the drain current, as shown in Figure S6 (Supporting Information).

Notably, the deterioration of the silver drain electrode after the measurement is visible in optical microscopy images (Figure S7, Supporting Information). This also applies to Cr electrodes which initially, i.e., in the first scan, exhibit the highest ON currents, but then degrade by 33% upon six repeated scans. We characterized the surface of our Cr electrodes after electrode patterning, but prior to perovskite deposition by X‐ray photoelectron spectroscopy (XPS, Figure S8, Supporting Information) and detected the signatures of chromium oxide and hydroxide in Cr 2*p*_3/2_ signal. No signature of metal oxidation states was found in Au, Pt and Ag samples (Figure S9, Supporting Information). The stability measurements of devices with CrO_x_ films of different thickness deposited on top of the Cr electrodes show that *I*_D_ drops faster when the chromium oxide thickness increases (Figure S10, Supporting Information). Interfacial reactions between halide perovskites and transport layer transition metal oxides (e.g., MoO_x_,^[^ ^33^ ^]^ TiO_x_,^[^ ^34^ ^]^ NiO_x_,^[^ ^34^, ^35^ ^]^ ZnO_x_,^[^ ^36^ ^]^ and CrO_x_^[^ ^37^, ^38^ ^]^) were widely reported for solar cells.^[^ ^39^, ^40^ ^]^ Therefore, it is likely that the interfacial degradation relevant in the case of Cr electrodes involves the surface chromium oxide layer. For high work function electrodes, electron injection at the source electrode becomes more difficult due to the high Schottky barrier at the M–S interface, as shown by the low *I*_D‐max_ in the first few cycles of transfer measurements performed on Pt and PFBT‐Au based devices (Figure 2b,c). The transfer curves of Au electrode‐based FETs (Figure 2a) are relatively stable upon repeated cycling and only show a small current build‐up. For Pt and PFBT‐modified Au the current build‐up is more pronounced, and in the case of Pt, the channel current does not reach values comparable to Au electrodes.

**Figure 3:**
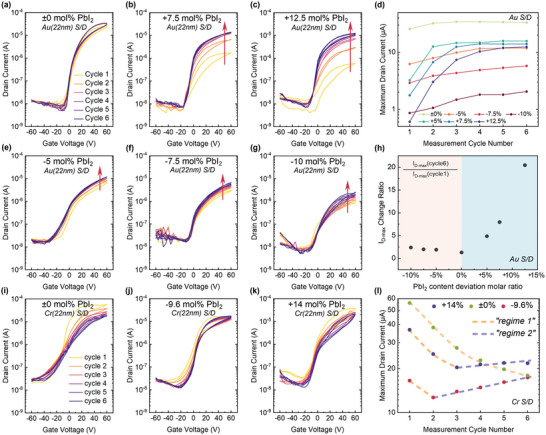
Transfer characteristics of Cs_0.05_FA_0.17_MA_0.78_PbI_3_ perovskite FETs with different PbI_2_‐to‐CsFAMAI ratio for six consecutive measurement cycles. a) Stoichiometric (with Au source and drain electrodes). b) +7.5 mol.% PbI_2_ (Au). c) +12.5 mol.% excess PbI_2_ (Au). e) −5 mol.% deficient PbI_2_ (Au). f) −7.5 mol.% deficient PbI_2_ (Au). g) −10 mol.% deficient PbI_2_ (Au). d) *I*_D‐max_ change to cycle number history for different compositions (for Au devices). h) Factor of *I*_D‐max_ increase versus PbI_2_ stoichiometry deviation. I_D‐max_ change ratio is the ratio between *I*_D‐max_ at cycle n and *I*_D‐max_ at cycle 1. Transfer characteristics of devices in Cr source and drain electrodes with different PbI_2_‐to‐CsFAMAI ratio for six consecutive measurement cycles are shown in i) stoichiometric (with Cr electrodes); j) −9.6 mol.% deficient PbI_2_ (Cr); k) +14 mol.% excess PbI_2_ (Cr); l) *I*_D‐max_ change (in log scale) to cycle number history for different compositions (for Cr electrode devices), where the current change could be categorized into two regimes. Regime 1: current decrease; Regime 2: current increase after the initial drop.

In the following we therefore focus on devices with gold electrodes and attempt to thoroughly investigate the current build‐up effect by intentionally altering the ionic defect environment of the Cs_0.05_FA_0.17_MA_0.78_PbI_3_ film. To do this, we vary the PbI_2_‐to‐CsFAMAI ratio in the precursor solution and then conduct charge transport stability measurements as a function of composition. The evolution of the transfer curves of these samples is shown in **Figure** **3**. Device with a stoichiometric composition exhibit relatively stable characteristics with *µ*_e,FET_ of 0.26 cm^2^ V^−1^ s^−1^ here (*µ*_e,FET_ values are listed in Figure S11, Supporting Information). In contrast, devices with non‐stoichiometric compositions show lower on‐current (<10 µA) and *µ*_e,FET_ (<0.02 cm^2^ V^−1^ s^−1^) in the initial cycle for both PbI_2_ excess and deficient samples. This suggests that the excess A‐site cation iodides (CsI/MAI/FAI) or PbI_2_ leftovers in the film significantly hinder the charge transport in these perovskite FETs. It is worth noting that both the off‐current and turn‐on voltages remain at similar values for all the compositions, suggesting that such stoichiometric changes (PbI_2_ molar deviation ratio from −10 to +12.5 mol.%) do not significantly modify the bulk conductivity.

In comparison with the small magnitude of change of transfer curves (*I*_D‐max_ increased by 30% after six cycles) in stoichiometric samples, the non‐stoichiometric samples exhibit a stronger current build‐up effect with an increase of on‐current by up to 2000% after repeated cycling. Notably, the PbI_2_ excess samples show a more pronounced current build‐up effect than PbI_2_ deficient samples. These trends are clearly depicted in plots of *I*_D‐max_ versus the number of cycles for the different compositions (Figure 3d) and plots of the factor by which *I*_D‐max_ increases versus PbI_2_ stoichiometry deviation (Figure 3h). The current build‐up effect is reversible if the devices are at rest in the dark for a certain time (Figure S12, Supporting Information). Notably, if we compare the increase in the absolute current values (Figure S13, Supporting Information), the PbI_2_ excess sample exhibits the highest increase and the PbI_2_ deficient sample shows the lowest. Devices that are overly bias‐stressed suffer from degradation and exhibit decaying current after ≈20 transfer sweeps regardless of the stoichiometry (Figure S14, Supporting Information). We also measured the devices at low temperature and found that the repeat‐cycle induced mobility increase is only pronounced at high temperatures (≈300 K) and suppressed at low temperatures (Figure S15, Supporting Information).

Previous research has suggested that the dominant defects in PbI_2_‐deficient samples are iodine interstitials $I_{i}^{-}$ ^[^ ^41^, ^42^ ^]^ while PbI_2_‐excess samples are $V_{I}^{+}$.^[^ ^41^, ^43^ ^]^ Our BGBC devices all exhibit robust *n*‐type charge transport, indicating that *p*‐type doping effect expected from $I_{i}^{-}$ is weak. However, in the case of PbI_2_‐deficient samples, we observe a tendency for slightly more positive turn‐on voltages potentially indicating some *n‐*type doping (Figure 3) and the small current level before and after bias may also be due to the background *p*‐type doping effect caused by the excess CsI/FAI/MAI. This is also consistent with our observation that top‐gate, bottom‐contact devices prepared under PbI_2_‐deficient conditions exhibit *p*‐type FET operation with low mobilities (Figure S16, Supporting Information). The reason for this dependence on device geometry is however not clear at present.

In contrast to the more robust gold electrodes, FETs fabricated with chromium electrodes and different PbI_2_‐to‐CsFAMAI ratio exhibit a complex non‐monotonic trend, as shown in Figure 3i–l. The device fabricated with the stoichiometric composition exhibits a continuous decrease in *I*_D‐max_ over six repeated runs, as shown in Figure 3i,l, similar to the results shown in Figure 1e for Cr. In contrast, the non‐stoichiometric samples show a drop in I_D‐max_ over the first 2‐3 cycles (regime 1), followed by stabilization and even a small increase upon further cycling (regime 2). As previously discussed, the trend observed in regime 1 is likely due to metal contact degradation caused by the low work function of chromium and the instability of chromium oxide species. This degradation appears to be present in all of the samples during their initial measurement cycles and cannot be avoided. The phenomena observed in regime 2 in the non‐stoichiometric samples is likely due to similar processes that lead to the current build‐up effect in Au devices. However, the stoichiometric sample (without excess species) does not show any increase in *I*_D‐max_ during the complete cycle of measurements. These results suggest that both contact degradation and current build‐up effects co‐exist in low work function electrode devices.

**Figure 4:**
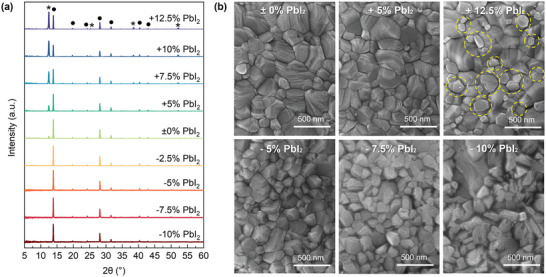
XRD and SEM characterizations of Cs_0.05_FA_0.17_MA_0.78_PbI_3_ perovskite samples with different PbI_2_ stoichiometries. a) XRD spectra for −10% molar deficient to +12.5% molar excess PbI_2_ samples. Characteristic peaks of PbI_2_ and perovskite are marked by asterisk (*) and black circle (●). b) SEM images showing the top surfaces of samples in stoichiometric composition, +5% molar excess PbI_2_, +12.5% molar excess PbI_2_, −5% molar deficient PbI_2_, −7.5% molar deficient PbI_2_ and −10% molar deficient PbI_2_. PbI_2_ hexagonal flakes are highlighted by the dashed circle on the image.

To better understand the dependence of electrical instabilities on precursor stoichiometry we examined the impact of precursor stoichiometry on phase composition and film morphology by X‐ray diffraction (XRD) and scanning electron microscopy (SEM). In excess‐PbI_2_ compositions, the presence of crystalline PbI_2_ was clearly identified by the characteristic PbI_2_ (001) peak at 12.5° (**Figure** **4**a) and the bright hexagonal PbI_2_ flakes^[^ ^44^, ^45^, ^46^ ^]^ on top of polycrystalline perovskite grains (SEM image Figure 4b, marked in yellow circles). Deficient‐PbI_2_ compositions show no signature of crystalline MAI^[^ ^47^ ^]^ (or FAI) in XRD but exhibit a more irregular surface morphology with a smaller grain‐size. This implies that excess PbI_2_ tends to form a separate phase,^[^ ^48^ ^]^ while excess MAI and FAI incorporate into perovskite lattice or grain boundaries.^[^ ^49^ ^]^ The XRD patterns for both PbI_2_‐excess and deficient samples show a small shift of the perovskite (110) peak by 0.02° toward smaller scattering angles (Figure S17, Supporting Information), which may indicate slight lattice expansion.

**Figure 5:**
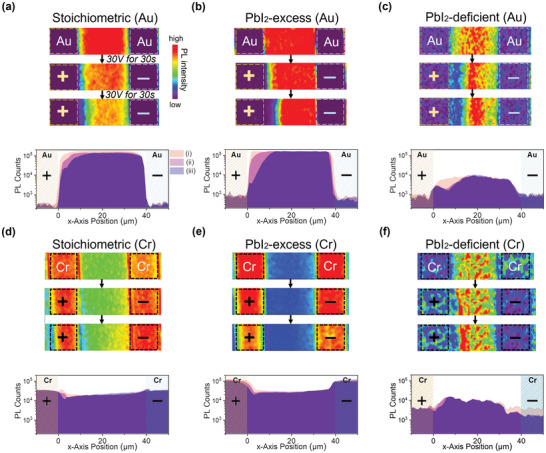
Photoluminescence response of Cs_0.05_FA_0.17_MA_0.78_PbI_3_ perovskite under a DC bias. a) Stoichiometric sample with Au contacts. b) PbI_2_‐excess sample (+12.5 mol.% excess PbI_2_) with Au contacts. c) PbI_2_‐deficient sample (−7.5 mol.% deficient PbI_2_) with Au contacts. The apparent hump in the PL profile close to the positive electrode after biasing is a feature exhibited only in this region and was not observed at other locations along the electrode edge (see Figure S20, Supporting Information). d) Stoichiometric sample with Cr contacts. e) PbI_2_‐excess sample (+12.5 mol.% excess PbI_2_) with Cr contacts. f) PbI_2_‐deficient sample (−7.5 mol.% deficient PbI_2_) with Cr contacts. In each panel, PL images are shown from three stages: i) a pristine film with no bias applied, ii) the same film that has been subjected to a 30 s DC bias of *V*_DS_ = +30 V, and iii) the same film that has been subjected to an additional 30 s of DC bias with *V*_DS_ = +30 V. Below the images are Y‐axis averaged PL intensity profiles for the three stages, using the same Y‐scale for all the spectra. To enhance the contrast of the color mapping, each panel has a unique PL intensity color scale. The channel length is 40 µm and the channel width is 1 mm.

To track the motion of ionic defects under electrical bias, we attempted to correlate the observed FET operational instabilities with photoluminescence (PL) imaging on the FET channels fabricated from Au and Cr electrodes. For this, we used very similar biasing conditions and applied a S–D bias of *V*_DS_ = +30 V for 30 s, imaged the channel, then applied the bias for a second cycle, and afterward reimaged the channel. **Figure** **5** shows the corresponding PL images for stochiometric, PbI_2_‐rich, and PbI_2_‐deficient films with Au and Cr electrodes. The corresponding PL intensity profiles (averaged along the y‐axis) of S–D channel are shown below the PL images. The comparatively low initial PL of PbI_2_‐deficient film (Figure 5c,f) suggests high defect density of the film,^[^ ^50^ ^]^ possibly due to a large number of interstitial iodine $I_{i}^{-}$, which is known to suppress PL emission.^[^ ^3^, ^51^ ^]^ The higher PL intensity in PbI_2_‐excess films (both Au and Cr, before or after the bias) could also manifest their low density of interstitial iodine $I_{i}^{-}$ when comparing to the stoichiometric and PbI_2_‐deficient films (see the detailed comparison of PL intensity between different samples in Figure S18, Supporting Information).

In the Au electrode samples (Figure 5a–c), a region with strongly quenched PL emerges near the positive Au electrode after S–D biasing for 30 s, and this region grows larger upon further biasing for 30 s. This effect is partially reversible when the device rests for 8 min (Figure S19, Supporting Information). In contrast, any PL quenching near the positive electrode upon biasing is much weaker in the Cr samples. Another key difference between Au and Cr samples is that, when comparing the PL intensity on top of the Au and Cr electrodes, the stoichiometric and PbI_2_‐excess devices exhibit a brighter PL signal on top of the chromium electrodes than in the channel, while in the case of gold, the PL signal detected on top of the electrodes is strongly reduced compared to the channel region. In the PbI_2_‐deficient samples (both Au and Cr), the PL signal is generally weaker, but there is a noticeable PL brightening in the middle part of the channel (Figure 5c,f;or more clearly in Figure S18e,f, Supporting Information). The Cr stoichiometric sample also shows a slight PL brightening in the region away from the positive Cr electrode (Figure 5d; or more clearly in Figure S18b, Supporting Information). The strong suppression of the PL on top of the Au electrodes is likely due to efficient extraction of photogenerated holes from the perovskite film into the Au contacts.

The quenching of the PL around the positive Au electrode is consistent with previous studies^[^ ^3^, ^7^, ^52^, ^53^, ^54^, ^55^, ^56^ ^]^ and has been studied extensively. Several mechanisms have been proposed, most involve the presence of negative iodide species near the positive electrode, such as the separation of Frenkel defect pairs $V_{I}^{+}-I_{i}^{-}$ ^[^ ^3^ ^]^ or the anodic oxidation of iodine gas (2I^−^ → I_2_ + 2e^−^) and formation of PbI_2_.^[^ ^55^ ^]^ The compensation of negatively charged point defects,^[^ ^3^ ^]^ such as iodide interstitials ($I_{i}^{-}$), by positively charged iodine vacancies migrating from the positive electrode may be responsible for the noticeable PL brightening in some low PL films in the middle part of the channel that occurs upon biasing (Figure 5c,d,f; or more clearly in Figure S18b,e,f, Supporting Information). As the concentration of unreacted MAI/FAI in the PbI_2_‐excess sample is expected to be lower than in the PbI_2_‐stoichiometric sample, the PbI_2_‐excess Au sample should exhibit less PL quenching from $I_{i}^{-}$. It is possible that other PL quenching species are contributing to the wider PL quench region observed in the PbI_2_‐excess Au sample.

The above mechanism may not completely explain the dependence of the degree of PL quenching on the choice of electrodes, which also suggests that other electrochemical processes at the positive electrode might also be involved. It has been shown that at positive potentials Au can be oxidized in the presence of iodide species leading to the electromigration of Au into the perovskite layer.^[^ ^7^, ^10^ ^]^ Interstitial ${\mathrm{Au}}_{i}^{+}$ as *n*‐type dopant or the formation of antisite Au_Pb_ as trap states^[^ ^8^ ^]^ are likely to trap the photogenerated holes and result in the PL quenching. In contrast, the expected high diffusion barrier for *n*‐type dopant ${\mathrm{Cr}}_{i}^{2+}$ may hinder its diffusion into the perovskite lattice^[^ ^8^ ^]^ thereby causing less PL quenching. Based on the preceding discussion, we believe that the overall PL intensity's dependence on composition is primarily due to the presence of unreacted FAI/MAI, while the PL quenching may be caused by the migration of both $I_{i}^{-}$ and extrinsic metallic ions, with the latter being particularly relevant for the electrode‐dependent PL quenching effect.

**Figure 6:**
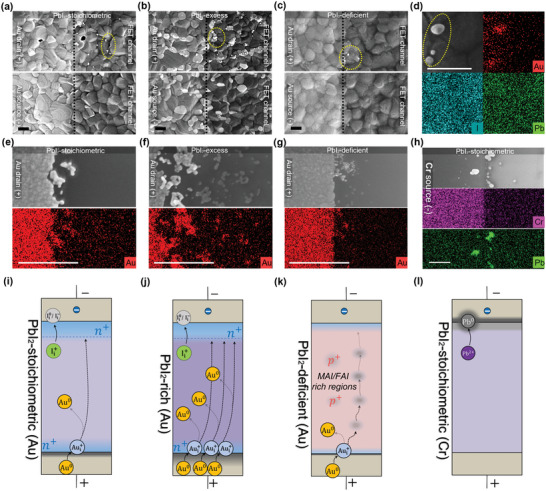
SEM and EDX mapping analysis of source and drain electrode areas in Cs_0.05_FA_0.17_MA_0.78_PbI_3_ perovskite FET devices that have undergone long‐term bias‐stress testing. SEM images are taken near the drain/perovskite and source/perovskite edge for a) stoichiometric, b) PbI_2_‐excess, and c) PbI_2_‐deficient films with Au electrodes, respectively. The perovskite over buried Au electrodes appears brighter than the channel area, and the electrode edges are marked by dashed lines in each image. In each image, bright nanoparticles found on top of the perovskite grains are highlighted by dot circles. d) EDX mapping analysis of Au, I, and Pb elements for the highlighted bright particles. SEM images of the exposed Au drain electrode edges in the devices after removing the perovskite layer by DMF‐wash along with the corresponding EDX mapping images of Au are shown for e) stoichiometric, f) PbI_2_‐excess, g) PbI_2_‐deficient films. h) SEM image and EDX mapping of Cr and Pb near the exposed Cr source electrode for a stoichiometric Cr device after bias‐stress testing and DMF‐wash. Schematics illustrating defect formation and migration in the different device cases are presented in i) PbI_2_‐stoichiometric composition with Au electrodes, j) PbI_2_‐excess composition with Au electrodes, k) PbI_2_‐deficient composition with Au electrodes, and l) PbI_2_‐stoichiometric composition with Cr electrodes. *N*‐type and *p*‐type doping regions are marked in blue and red, respectively, while degraded electrode edges are marked in grey. All SEM and EDX mapping images have a scale bar of 200 nm.

To further investigate the physical cause of the current build‐up effect and the PL quenching, we conducted electron microscopy analysis on Au electrodes with different stoichiometry. These devices were subjected to a harsher bias‐stress protocol involving 150 transfer sweeps to create sufficient morphological changes that could be detected using SEM (resolution: ≈5 nm). **Figure** **6**a–c shows SEM images taken near the drain/perovskite and source/perovskite edge for stoichiometric, PbI_2_‐excess, and PbI_2_‐deficient films with Au electrodes. The electrode region and electrode edge are visible because the perovskite deposited on top of the Au exhibits higher brightness. In the channel area near the drain electrode edge, there are bright particles on top of the perovskite grains, as highlighted by the dot circles in the image. EDX mapping in Figure 6d reveals that these particles are made of Au. The high brightness and solid spherical geometry suggest that these particles are most likely to be metallic Au(0) rather than ionized Au states. Similar Au particles near a positively biased electrode were also reported by Kerner et al.^[^ ^7^ ^]^ The PbI_2_‐excess film has the highest number of particles, which extend far into the channel (Figure 6b). Most of these particles are located on bright hexagonal PbI_2_ grains. The stoichiometric film has fewer particles, most of which are located on grain boundaries (Figure 6a). The PbI_2_‐deficient film shows the smallest number of Au particles, which only appear at the drain electrode edge (Figure 6c). No Au particle was observed near the source electrode edge for all films. The Au particles were also observable in a film that is only moderately biased for ten transfer sweeps (Figure S21, Supporting Information).

To confirm that these Au particles originate from the positive Au drain electrode, we washed away the perovskite layer from the similarly bias‐stressed devices and investigated the exposed Au electrode surface. Figure 6e–g shows the SEM images of the exposed Au drain electrode edge and the corresponding EDX mapping images of Au for stochiometric, PbI_2_‐excess, and PbI_2_‐deficient films. Surprisingly, we found significant degradation at Au drain where Au grains at the edge break into pieces into the channel. The degradation is most severe for PbI_2_‐excess film where Au pieces span over 400 nm wide from the drain electrode edge (Figure 6f). PbI_2_‐deficient film shows the least pronounced degradation as evidenced by the small size of Au clusters and relatively smooth drain edge (Figure 6g).

In Figure S22 (Supporting Information), the corresponding SEM images of the source electrodes show that there is no such degradation and gold cluster formation at the edge. However, at the source, the electrode surface near the edge appears darker than the interior electrode area in the stoichiometric film. The PbI_2_‐excess film exhibits an even wider dark region, while the PbI_2_‐deficient film is mostly bright. This dark region is not visible in the positively biased or unbiased electrodes (as shown in Figure S23, Supporting Information). The width of the dark belt on the source electrode is ≈1.5 µm for the PbI_2_‐excess film and ≈0.9 µm for the stoichiometric film (Figure S23d,e, Supporting Information). Apart from Au signal, EDX mapping suggests no noticeable difference of Pb and iodine distribution between different films (Figures S24 and S25, Supporting Information). The dark belt is less likely due to metallic nanoparticles as all the Au and Pb particles observed in this measurement are very bright. Although we did not perform other elemental analysis methods such as XPS due to the small size of the belt region, the aggregation of iodine species near a cathode has been confirmed by Kerner and Xu et al.,^[^ ^11^, ^12^, ^57^ ^]^ where they suggest positively charged iodine interstitial $I_{i}^{+}$ can migrate to a cathode and be reduced to lattice iodide I^−^. Higgins et al. also observed the trace signal of Au‐halide complexes over negatively biased Au electrode by ToF‐SIMS.^[^ ^58^ ^]^ Hence, $I_{i}^{+}$ seems to be a possible species to be reduced at the negative Au source and the dark belt could be a region rich in the reduction products of $I_{i}^{+}$, such as I^0^ and lattice I^−^, which might form metal‐halide complexes with Au. We could not exclude MA^+^ or FA^+^ as they can also migrate to the negative electrode as observed by Yamilova et al. also using ToF‐SIMS.^[^ ^55^ ^]^ Another effect that was observed is that in both biased and unbiased devices, the grain size of Au in the electrode pads covered by the PbI_2_‐deficient perovskite appears to be larger than in the other films, as shown in Figures S22 and S23 (Supporting Information). The reason for this effect is currently not clear.

Based on the above evidence, we can confidently conclude that Au at the positively biased drain electrode becomes oxidized and migrates into the bulk of the perovskite material in the form of Au ions. Some of these Au ions are then reduced back to Au(0) particles due to the presence of electrons injected from the source. The effect of PbI_2_ stoichiometry on this process is also evident: excess MAI/FAI slows down the degradation and migration of Au at the drain, while excess PbI_2_ accelerates both. This acceleration effect of PbI_2_ is further supported by the significant amount of Au particles that were observed on a pure PbI_2_ film after bias‐stress (Figure S26, Supporting Information).

We performed a similar analysis on a Cr electrode device (PbI_2_‐stoichiometric) which was overly bias‐stressed for 200 transfer sweeps. Unlike with Au devices, we did not observe any metallic particles on the surface of the perovskite layer (Figure S27, Supporting Information). After washing the perovskite layer, we used SEM and EDX to examine the exposed electrodes. We found that the positively biased Cr drain electrode was undamaged (Figure S28b, Supporting Information), but there were many bright particles on the edge of negatively biased Cr source electrode (Figure S28c, Supporting Information). EDX also showed that there was no diffusion of Cr into the channel at the drain electrode (Figure S29, Supporting Information), possibly due to the high diffusion barrier of ${\mathrm{Cr}}_{i}^{2+}$ in perovskite lattice.^[^ ^8^ ^]^ EDX mapping in Figure 6h shows that the particles at the Cr source edge are made of Pb. The formation of metallic Pb(0) near a negatively biased electrode has been reported by Kerner et al. using XPS.^[^ ^5^ ^]^ The solid spherical shape and high brightness of the particles here suggest that they are also likely Pb(0) rather than other oxide states of Pb. These observations indicate that the degradation in Cr electrode devices occurs primarily at the negatively biased source electrode, where Pb^2+^ is reduced to Pb(0).

## 3 Discussion

Some of the observed dependence of FET characteristics on film composition, such as the generally low FET current observed under PbI_2_ deficient conditions, could directly reflect a dependence of the FET mobility on stoichiometry‐dependent defect concentrations in the channel. It is also possible that some of the observed current build‐up effect could be due to the migration of these stoichiometry‐dependent defects toward the electrodes, potentially due to a “defect cleaning” effect in the channel. However, the observed strong dependence of the FET instabilities upon repeated device cycling on the choice of electrodes suggests that the instabilities are more likely to be caused by biasing‐induced electro‐chemical processes at the electrodes leading to defect migration into the channel. This could lead to charge trap formation or doping of the channel. If the migrating defect generated at the electrode interface is an *n*‐type dopant, for example, this might compensate electron trap states in the channel and could lead to an increase in the FET current upon repeated cycling. Alternatively, the electrochemical process on the electrode could lead to changes in electron injection/extraction barrier at the source and drain electrodes, which may limit the ON current in *n*‐type FETs with relatively high work function electrodes. Because the Schottky barrier formed at the source electrode is reverse biased in the ON state, while the corresponding barrier at the drain electrode is forward biased, the increase of FET ON current upon repeated cycling could be due to an improvement of the electron injection from the negatively biased source electrode into the perovskite layer.

We have clear evidence from the SEM/EDX results that in the case of gold electrodes Au^+^ migrates from the positive drain electrode toward the negative source electrode, as illustrated in Figure 6i. Au^+^ is expected to be an *n*‐type dopant and could therefore be responsible for the observed current build‐up effect, either by doping the channel region *n*‐type or by *n*‐type doping the contact interfaces and reducing the width of the depletion layer affecting electron injection (Figure S30, Supporting Information). We note that in our SEM/EDX experiments we did not resolve Au^+^ diffusion directly due to its low concentration and limited elemental resolution of EDX detector, we only observe it indirectly when it becomes reduced to Au^0^ and forms clusters. Thermodynamically, this is more likely to occur in regions of the film that are more highly *n*‐type doped, i.e., close to the positive electrode, but our measurement does not exclude the possibility that gold diffuses all the way across the channel and improves the electron injection at the source electrode.

To further validate the role of Au^+^ doping in the current build‐up effect observed with gold electrodes, we attempted to dope the material by adding different molar ratios of gold iodide (0, 0.5, and 2 mol.%) and performed repeated long‐cycle FET transfer characteristics measurements. Our results, shown in Figure S31 (Supporting Information), indicate that the 0.5 mol.% AuI‐doped sample exhibits a significant increase in drain current of ≈100% upon cycling, compared to the pristine sample which did not show any increase. The increase in drain current was even more pronounced in the sample doped with 2 mol.% AuI, with an increase of ≈400% to itself at cycle seven. These results are consistent with the current build‐up effect being caused by the migration of Au^+^ defects in the film. Kerner et al. have very recently observed a similar migration of Au^+^ interstitials from an Au anode in a MAPbI_3_ perovskite diode, which enhances *n*‐type doping of the perovskite layer.^[^ ^10^ ^]^ Pospisil et al. also observed improved charge carrier injection in a two‐terminal MAPbBr_3_ device with AuI‐modified Au electrode.^[^ ^59^ ^]^

This proposed model can also explain the observed dependence of the current build‐up effect on stoichiometry. As shown in Figure 6j, the accelerated migration of Au in PbI_2_‐excess conditions that is apparent in the SEM/EDX images could facilitate more pronounced *n*‐type doping resulting in a higher increase in *I*_D_ compared to PbI_2_ stoichiometric or deficient conditions. In contrast, Figure 6k illustrates that under PbI_2_ deficient conditions there is either less Au migration into the channel or the excess of FAI and MAI slows down the migration of Au and limits the current build‐up effect. The background *p*‐type doping of excess FAI/MAI also limits *I*_D_ of these devices to be much smaller than in other samples (Figure S13, Supporting Information).

On the other hand, low work function metals, whose work functions are close to the conduction band minimum (CBM) of MAPbI_3_ (≈−4 eV^[^ ^60^ ^]^), can already facilitate good electron injection without the need for interfacial doping processes. However, as Pb is more electronegative than these metals,^[^ ^61^ ^]^ Pb^2+^ ions are more likely to be reduced than the electrode metal ions themselves. In the case of Cr, as evidenced directly by SEM/EDX and illustrated in Figure 6l, Pb(0) forms at the negatively biased source electrode. This creates deep *p*‐type traps ($V_{\mathrm{Pb}}^{2-}$) in the perovskite near the source electrode, which are expected to deteriorate electron injection. This, combined with the instability of surface oxides mentioned in Figure S10 (Supporting Information), could explain the current decay observed in Cr devices.

## 4 Conclusion

In conclusion, we have studied the influence of source–drain electrode work function and film stoichiometry on the performance and operational stability of *n*‐type CsFAMAPbI_3_ perovskite FETs. Under prolonged S–D bias, we found that the ON‐state channel current and FET mobility could increase or decrease, depending on the work function of the contacts. While the highest ON currents can be achieved with low work function Cr electrodes, we found these to have limited stability and the ON current decreased by almost an order of magnitude upon repeated device cycling. Similar device degradation was also observed for other low work function electrodes. On the other hand, higher work function electrodes exhibited an improvement and current build‐up during repeated cycling. Devices with Au electrodes reached ON currents close to those of Cr devices upon repeated cycling. The operational stability was also found to be strongly dependent on film composition. Stoichiometric samples exhibited the best operational stability, while samples prepared with excess and deficient PbI_2_ composition and high work function electrodes required repeated cycling to reach a higher ON currents. These operational instabilities as a function of metal electrode and sample composition were correlated with the quenching of the PL near the positive electrode that could be observed under the same biasing conditions, which we found to be more pronounced for Au than for Cr electrodes. The PL quenching mechanism has been investigated extensively in the literature and has been attributed to lateral migration of iodide defects at the positive electrode. Our results correlating the PL quenching on the choice of metal electrodes suggest an additional PL quenching mechanism involving the migration of extrinsic metallic ions, such as Au^+^. Our elemental mapping analysis showed that the bias‐driven degradation and migration of Au at the drain electrode may contribute to the increase in ON current in Au samples through *n*‐type doping of the channel or the metal–perovskite interface. With the diffusion of Au ions potentially being affected by excess PbI_2_ or the presence of excess FAI/MAI, the device operational stability thereby shows a dependence on sample composition. In contrast, the decay of ON current in Cr samples is caused by the formation of Pb(0) at the negative electrode due to the degradation of the perovskite. These findings shed light on how bias‐induced migration of extrinsic metallic ions impacts charge transport at a device level, revealing the widespread nature of this phenomenon when perovskite is in direct contact with metals. This underscores the crucial role of careful metal selection and precise composition control for the successful design of durable and reliable perovskite‐based optoelectronic devices.

## 5 Experimental Section

### Materials

Formamidinium iodide (FAI) (99.99%), Methylammonium iodide (MAI) (99.99%) were purchased from Greatcell Solar Materials. Ultra‐dry lead iodide PbI_2_ (99.999%) and gold monoiodide (AuI) (99%) were purchased from Alfa Aesar. Cesium iodide (CsI) (99.999%), N,N‐dimethylformamide (DMF) (99.8% anhydrous), dimethyl sulfoxide (DMSO) (99.9% anhydrous), chlorobenzene (99.8% anhydrous), Polyethylenimine, 80% ethoxylated (PEIE) solution and 2,3,4,5,6‐Pentafluorothiophenol (PFBT) (97%) solution were purchased from Sigma–Aldrich.

### FET Substrate Fabrication

SiO_2_ (300 nm SiO_2_ on n++ silicon) or glass substrates are pre‐patterned with FET channel patterns via photolithography. Metal electrodes were deposited by thermal evaporation in high vacuum (<2 × 10^−6^ mbar). Cr (4 nm) at an evaporation rate 0.5 Å s^−1^ was deposited to serve as an adhesion layer between SiO_2_ and other metals. Then, 22 nm of metal (Cr, Au, Ag, Ti, Au, and Pd) was deposited at a rate of 0.5–0.8 Å s^−1^. For Cr–CrO_x_ films, an additional layer of 1–5 nm of Cr was deposited in low vacuum (1 × 10^−3^ mbar). Substrates were then lifted‐off by N‐Methyl‐2‐pyrrolidone (NMP) and cleaned by acetone and isopropanol. PEIE and PFBT treatments were applied to Au substrates by the method described in Ref. [23] Sputter coated Pt electrodes were prepared in Ar atmosphere (5 × 10^−4^ mbar) in a chamber that had been pumped to a high vacuum before (<4 × 10^−6^ mbar). A 5 nm NiCr adhesion layer was deposited between the substrate and the 30 nm Pt electrode. RF power of 50 W was used to sputter coat, affording a deposition rate of 4 Å s^−1^ for Pt and 2 Å s^−1^ for NiCr.

### Perovskite Thin Film Deposition

All the operations in this section were carried out in an N_2_ filled glovebox (O_2_ < 0.5 ppm, H_2_O < 0.5 ppm). FAI, MAI, CsI and PbI_2_ were weighted and dissolved in a DMF–DMSO cosolvent (volume ratio DMF:DMSO = 4:1) to form a precursor solution of Cs_0.05_FA_0.17_MA_0.78_PbI_3_ in a concentration of 0.75 m. For samples with deficient and excess PbI_2_ content, the concentration of Cs_0.05_FA_0.17_MA_0.78_I was kept at 0.75 m, while the concentration of PbI_2_ was tuned to be (1 + x) × 0.75 m, where x% is the molar deviation ratio of PbI_2_ (x% ranges from −10% to +15%). The precursor was heated and shaken at 50 °C for 30 min to facilitate dissolving. The precursor (40 microlitre) was then dropped onto the substrates for spin coating at 5000 rpm for 40 s and chlorobenzene was dropped at the 8th second. The films were finally annealed at 100 °C for 30 min.

### FET Measurements

Transfer curve measurements were carried out on a Desert TTP4 probe station by an Agilent 4155C Semiconductor Parameter Analyzer. Samples were loaded into the probe station chamber and pumped to high vacuum (<10^−5^ mbar). Transfer sweeps were performed in pulsed mode, with pre‐stated pulse interval time *'t'* and voltage increment step *'s'* using the same integration time of 1 ms.

### XRD and SEM Measurements

Measurements were directly carried out on the FET samples (perovskite on SiO_2_ substrates) after electrical characterization. XRD measurements were collected in the Bragg‐Brentano geometry using a Bruker D8 Advance powder X‐ray diffractometer with Cu K*α* (*λ* = 1.54 Å) radiation. The setup operates in reflection, theta–theta mode (fixed sample) with a 2D strip detector. A beam mask of 0.6 cm was chosen to properly irradiate the films. A step size of 0.01° with time/step set to 0.15 s were used for the measurements. SEM imaging was carried out in high vacuum (<4 × 10^−6^ mbar) by a LEO GEMINI 1530VP FEG‐SEM using 20 kV acceleration voltage. EDX mapping was carried out by a Xplore30 EDS detector using 8 mm working distance. Ex situ SEM and EDX analysis were carried out on devices (PbI_2_‐stoichiometric, +30 mol.% PbI_2_, −20 mol.% PbI_2_) which were bias‐stressed for 150 transfer sweeps at +60 V *V*_DS_ and 0–+60 V *V*_GS_ (step 4 V). The perovskite layer was removed by dripping 200 µL DMF on the chip in a spin‐coating process at 5000 rpm for 40 s without further annealing or drying.

### Photoluminescence Imaging

Perovskite films for PL mapping measurements were spin coated on pre‐patterned glass substrates with FET patterns without gate electrode. PL measurements were performed using the same method and platform (WITec, Alpha 300 RAS confocal microscope) as described in Ref. [16] in which a fiber‐coupled 405‐nm continuous‐wave laser (Coherent, CUBE) was focused onto the sample using 40× objective and the average power of the laser at its focal point was 0.5 µW. The PL of the sample was collected in the reflection geometry from the same objective while the excitation laser beam from the reflection was blocked using a 415‐nm long‐pass filter. The collected PL spectrum was measured using a calibrated Si‐charge‐coupled device array detector (Andor, iDus BR‐DD) fitted to a monochromator. All movements of the stage were automated and controlled by WITec ScanCtr spectroscopy Plus software. All PL spectra were performed in an N_2_ atmosphere (O_2_ concentration <1%). The source–drain bias was applied using a Keithley 2400 source meter.

### UPS and XPS Measurements

XPS (AlK*α*, 1486.7 eV) and UPS (He I, 21.2 eV) spectra were collected in a Thermo Fisher Scientific Escalab 250Xi spectrometer. For XPS measurements, a pass energy of 10 or 20 eV, step size of 0.1 eV, spot size of 950 µm was used. For UPS measurements, a pass energy of 2 eV, step size of 0.01 eV, spot size of 2000 µm was used. An electrical bias of −5 or −20 V was applied on the UPS samples to record the secondary electron cutoff and extract the work function of the samples. The base pressure for XPS measurements was ≈5 × 10^−9^ mbar, and for UPS was 1 × 10^−8^ mbar.

## Conflict of Interest

The authors declare no conflict of interest.

## Author Contributions

Y.Z. conceptualized the idea, designed the experiments, fabricated the FET devices and performed charge transport measurements, ex situ SEM‐EDX measurements, analyzed the results and wrote the manuscript with inputs from H.S. and S.P.S.. R.S. and Y.Z. performed PL mapping measurements. D.H.L.T. and Y.Z. performed the UPS and XPS measurements. H.‐I.U. assisted Y.Z. in the ex situ SEM‐EDX measurements. D.H.L.T., S.P.S., and M.X. assisted Y.Z. in the fabrication of different metal contacts. A.U. and Y.Z. collected XRD data. A.U. and S.P.S. assisted Y.Z. in the initial optimization of mixed‐cation perovskite films. H.S. supervised the project. All authors discussed the work and revised the manuscript.

## Supporting information

Supporting Information

## Data Availability

The data that support the findings of this study are available from the corresponding author upon reasonable request.
