# Supplementary material for: Direct Observation of Contact Reaction Induced Ion Migration and its Effect on Non‐Ideal Charge Transport in Lead Triiodide Perovskite Field‐Effect Transistors
Source: Small. 2023 Jun 10;19(41):2302494. doi: 10.1002/smll.202302494 (PMC11475287; doi:10.1002/smll.202302494)
Supplement: Supplementary file 1 — Supporting Information [file SMLL-19-2302494-s001.pdf]

NANO · MICRO
small

Supporting Information

for *Small*, DOI 10.1002/smll.202302494

Direct Observation of Contact Reaction Induced Ion Migration and its Effect on Non-Ideal Charge Transport in Lead Triiodide Perovskite Field-Effect Transistors

Youcheng Zhang, Amita Ummadisingu*, Ravichandran Shivanna, Dionisius Hardjo Lukito Tjhe, Hio-Ieng Un, Mingfei Xiao, Richard H. Friend, Satyaprasad P. Senanayak and Henning Sirringhaus**

Supporting Information

Direct Observation of Contact Reaction Induced Ion Migration and its Effect on Non-Ideal Charge Transport in Lead Triiodide Perovskite Field-Effect Transistors

Youcheng Zhang, Amita Ummadisingu, Ravichandran Shivanna, Dionisius Hardjo Lukito Tjhe, Hio-Ieng Un, Mingfei Xiao, Richard H. Friend, Satyaprasad P Senanayak, Henning Sirringhaus**

Supplementary Note 1: Influence of gate bias on FET characteristics
Supplementary Note 2: Work function measurements for metal contacts
Supplementary Note 3: Manifestation of other low and high work function electrodes
Supplementary Note 4: Degradation of silver contacts
Supplementary Note 5: XPS measurements of electrode surfaces
Supplementary Note 6: Channel current decay in devices with Cr and CrO_x electrodes
Supplementary Note 7: Detailed transport characterization on samples with different stoichiometry under S-D bias
Supplementary Note 8: Observation of weak p-type transport in top-gated device
Supplementary Note 9: Detailed XRD characterization of stoichiometric and non-stoichiometric $Cs_{0.05}FA_{0.17}MA_{0.78}PbI_3$ films
Supplementary Note 10: Photoluminescence mapping analysis on biased Au and Cr devices with different stoichiometry
Supplementary Note 11: Ex situ SEM and EDX analysis on biased Au devices with different stoichiometry
Supplementary Note 12: SEM and EDX analysis of a pure-PbI_2 FET device after long term bias stress
Supplementary Note 13: Ex situ SEM and EDX analysis on biased Cr devices
Supplementary Note 14: Diagrams illustrating the potential changes in the band structure following interfacial n-doping
Supplementary Note 15: The effect of Au^+ doping

Supplementary Note 1: Influence of gate bias on FET characteristics

The transfer characteristics of bottom-contact, bottom-gate FET devices were repeatedly measured to probe the evolution of the drain current. To mitigate the trapping effect of mobile ions at the semiconductor-dielectric interface, we perform the transfer characteristics measurement in gate-pulsed mode, where the gate bias at each V_{GS} value is only applied for 1 millisecond, while a constant source-drain bias (+60V) is applied during the entire measurement cycle. It is shown in the main paper that repeated cycling induces an increase of channel current, which is referred to as "current build-up effect".

To decouple the effects on I_{DS} evolution of S-D bias from that of gate pulsing, we measured devices without any gate pulsing history, but subjected to a S-D bias before the measurement. Transfer characteristics of different FET devices pre-biased at V_{DS}=+60V for 2.5 to 4.5 minutes are shown in **Figure S1** (a). The pre-biased devices already show a maximum drain current ($I_{D\text{-}max}$) above ~20 μA, which is comparable to the $I_{D\text{-}max}$ obtained from devices that were not pre-biased, but subjected to repeated transfer sweeps. The pre-S-D bias time of these devices also approximately matches the duration of 6 short transfer measurement cycles (~3 mins). Therefore, we can mainly attribute the pronounced current increase in the repeated cycling measurements to the application of a constant S-D bias. We also observe larger hysteresis in the characteristics of devices which were biased for 3.5~4.5 minutes. If we keep measuring the pre-biased device for multiple cycles, the transfer curves barely changed, as shown in **Figure S1** (b). This suggests that 2.5 mins of S-D bias can build up the $I_{D\text{-}max}$ to a high level.

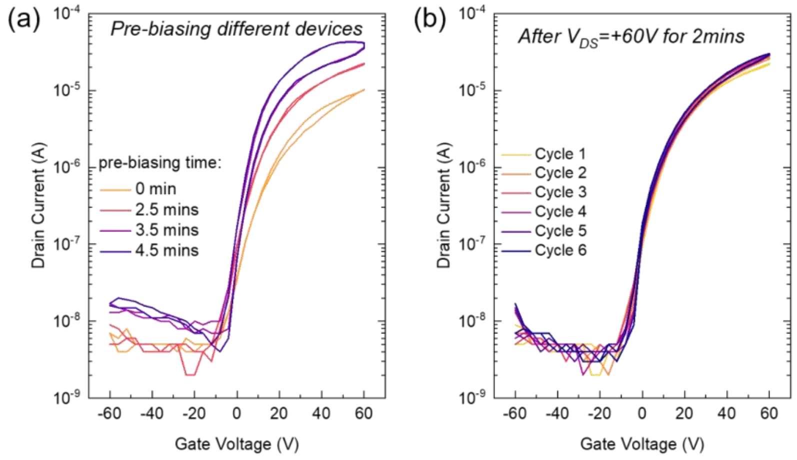

Figure S1 (a) Transfer characteristics of $Cs_{0.05}FA_{0.17}MA_{0.78}PbI_3$ (+5% molar extra PbI_2) devices which were pre-biased at V_{DS}=+60V, V_{GS}=0V for 0, 2.5, 3.5, 4.5 minutes (on separate devices); (b) Transfer characteristics of six consecutives cycles measured immediately after the 2.5 minutes' pre-S-D bias. Measurement parameters: s=4V, t=0.5s. Channel length L=100μm, channel width W=1 mm. V_{DS}=+60V, V_{GS} sweeps between -60V and +60V. Source and drain electrodes: 22 nm Au. Dielectric: 300nm of SiO_2.

Pulsed gate bias is applied via the silicon substrate which acts as a common bottom gate electrode. To assess the influence of gate biasing via a common gate on the other devices on the substrate that are not being measured, three devices on a same chip ($Cs_{0.05}FA_{0.17}MA_{0.78}PbI_3$ with +20% molar extra PbI_2) were consecutively measured without delay. Transfer characteristics of the devices using long-cycle measurement conditions are shown in **Figure S 2**. **Figure S 2** compares cycle 1 transfer characteristics from different devices. All the devices show a consistent $I_{D\text{-}max}$ value around ~5.5 μA. This suggests that the application of a gate voltage through a common gate has negligible effect on the performance of other devices which are not subjected to a source-drain bias.

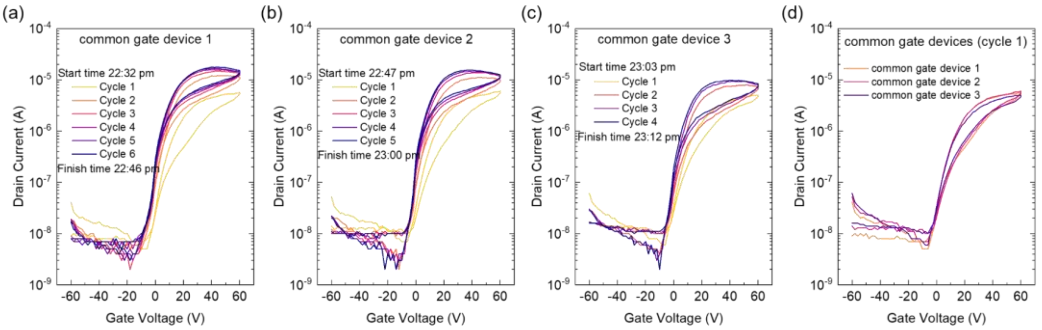

Figure S 2 Transfer characteristics of $Cs_{0.05}FA_{0.17}MA_{0.78}PbI_3$ (+20% molar extra PbI_2) devices consecutively measured using a common bottom gate. Measurement sequence: (a) device 1 (6 cycles) → (b) device 2 (5 cycles) → (c) device 3 (4 cycles). For each device, the start and finish measurement time of the day is marked on the graph. After finishing the measurement of one device, it took 1~3 minutes to move the probes to the next device. (d) Comparison of the cycle 1 transfer curves between device 1, 2, 3. Measurement parameters: s=2V, t=1s. Channel length L=100μm, channel width W=1 mm. V_{DS}=+60V, V_{GS} sweeps between -60V and +60V. Source and drain electrodes: 22 nm Au. Dielectric: 300nm SiO_2.

Supplementary Note 2: Work function measurements for metal contacts

The work function (WF) of different electrode materials was measured by ultraviolet photoelectron spectroscopy (UPS). This measurement could not be performed directly on the device electrodes due to the small electrode size compared to the large XPS/UPS spot size

(950µm for XPS and 2000µm for UPS). To reproduce the original surface condition of the metal electrodes with high fidelity on a large-area film for XPS/UPS, a plain SiO_2 substrate was processed in parallel during the thermal evaporation or sputtering of electrodes and experienced the same lift-off and cleaning process by NMP, acetone and isopropanol as the one used for the photolithographic electrode patterning process. The PEIE modification process was also comparable to that used for device fabrication. **Figure S 3** shows the UPS spectra of Pt, Au, Ag, PEIE-Au, Cr and CrO_x(1nm)-Cr films. -5V electrode bias was applied for the measurement of Pt, Au, Ag and PEIE-Au. The 5d band structure is clearly seen in the spectra of Pt and Au between 20 to 25 eV. For Cr samples, a higher bias of -20V was applied to improve signal quality. The value of cut off energy ($E_{cut-off}$) is the x-axis intercept obtained by the extrapolation of the secondary electron cut-off edge from the spectrum. The work function values extracted from the UPS measurements are listed in **Figure 2** (h).

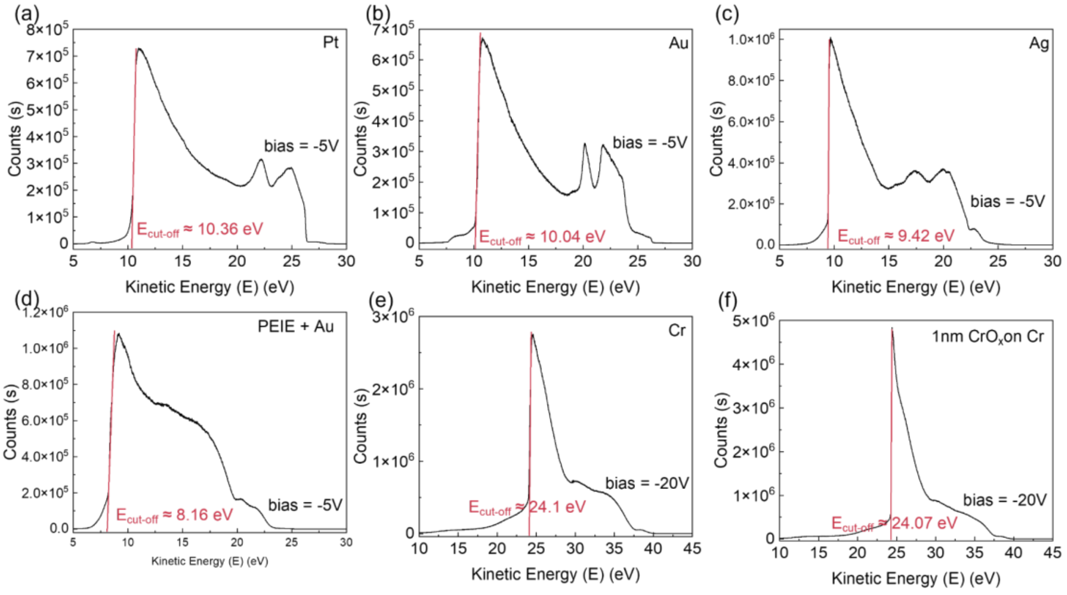

Figure S 3 UPS spectra of (a) Pt, (b) Au, (c) Ag, (d) PEIE modified Au, (e) Cr and (f) 1 nm CrO_x modified Cr. The cut-off energy and negative bias applied during the measurement are marked on each graph for the estimation of work function.

Our work function data is similar to the values reported in the literature. **Figure S 4** lists the literature work function values of the electrodes. Values from different reports show variations between 0.1 to 0.5 eV due to differences in measurement methods, sample conditions and crystalline orientation. We categorize the electrodes as low and high work function materials with respect to gold, as depicted by the blue and yellow background colours on the graph. Our results for Cr, Ag, Au, Pt and PEIE-Au are close to the literature values.

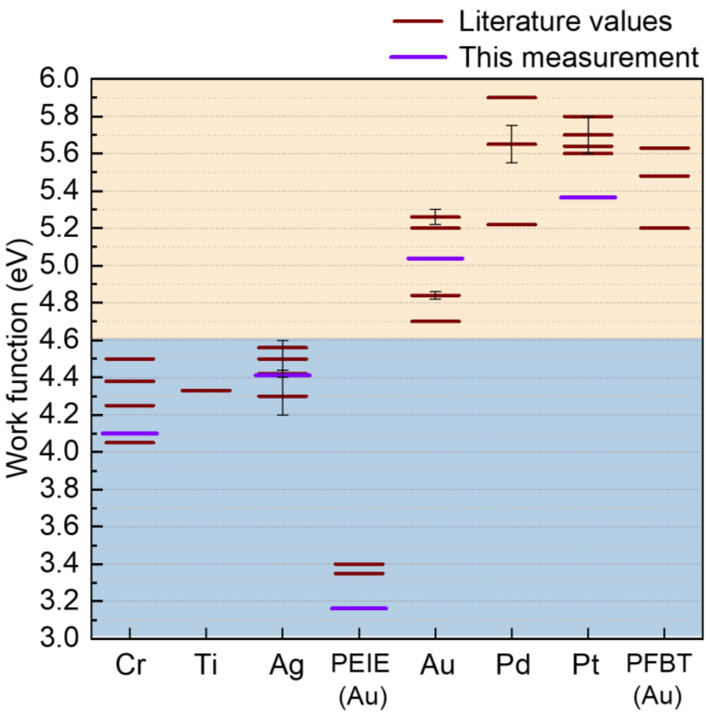

Figure S 4 Electrode work function values obtained from this measurement (violet line) and literature (brown line). Literature work function values include: metallic Cr [1,2], metallic Ti [3,4], metallic Ag [5], PEIE modified Au [6,7], metallic Au [3,5,7–10], metallic palladium (Pd)[3,5], metallic Pt [3,5] and PFBT modified Au [7,9–11].

Supplementary Note 3: Manifestation of other low and high work function electrodes

We further tested one more group of low and high work function materials as source and drain electrodes to validate our findings. **Figure S 4** shows that the work function values for metallic Ti and Pd are 4.33 eV and ~5.2-5.9 eV. The work function of Au(60%)-Pd(40%) alloy should be higher than the WF value of pure Au. Therefore, Ti can be categorized as low work function material and Au-Pd alloy can be taken as high work function material. We deposited 22 nm of Ti and 15 nm of Au-Pd electrodes by thermal evaporation. Stoichiometric $Cs_{0.05}FA_{0.17}MA_{0.78}PbI_3$ was spin-coated on these substrates by a standard procedure described in the methods section. **Figure S 5** shows the repeated short-cycle transfer characteristics of these samples. Device with Ti electrodes exhibited a continuous of drop of ON current, while device with Au-Pd electrodes exhibited pronounced current build-up effect. This result is consistent with our observations for other types of low and high work function electrode discussed in **Figure 2**.

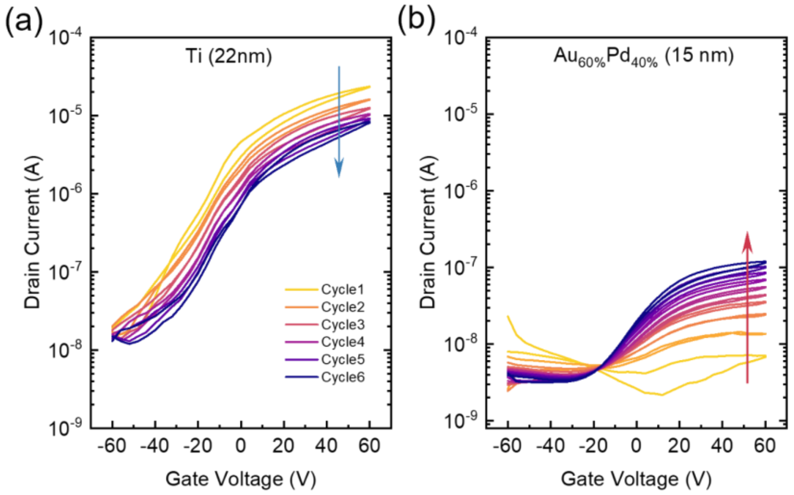

Figure S 5 Transfer characteristics for $Cs_{0.05}FA_{0.17}MA_{0.78}PbI_3$ perovskites with (a) 22 nm Titanium source and drain electrodes, and (b) 15 nm Au(60%)-Pd(40%) alloy source and drain electrodes. Measurement parameters: s=4V, t=0.5s. Channel length L=100µm, channel width W=1 mm. Ti and Au-Pd alloy are evaporated onto the pre-patterned SiO_2 substrates in high vacuum ($2*10^{-6}$ mbar). A layer of 4 nm Chromium was evaporated between SiO_2 and Au-Pd as an adhesion layer. Measurement parameters: s=4V, t=0.5s. Channel length L=100µm, channel width W=1 mm. V_{DS}=+60V, V_{GS} sweeps between -60V and +60V. Dielectric: 300nm SiO_2.

The FET mobility and threshold voltage values of the tested devices with different metal contacts are plotted in **Figure S 6**. Low work function electrode devices exhibit a drop of mobility and threshold voltage when cycled repeatedly, suggesting a p-doping process taken place during the bias-stress. High work function electrodes show an increase of FET mobility and no clear changing pattern for the threshold voltage.

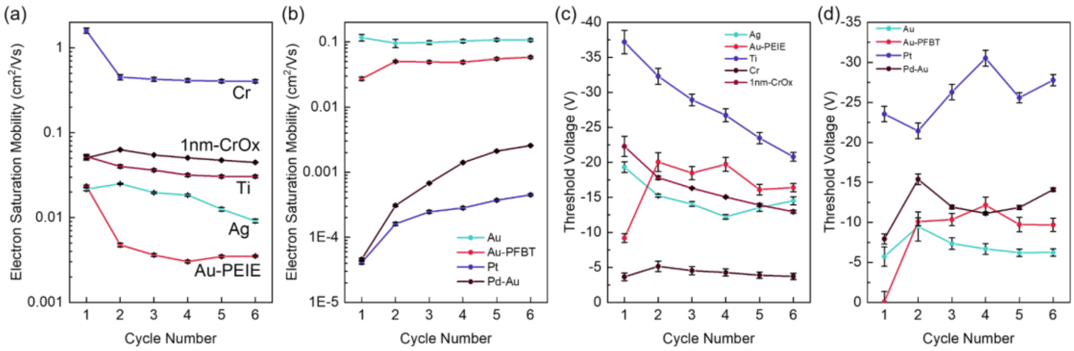

Figure S 6: Extracted FET mobility values as a function of cycle number for (a) low work function contacts and (b) high work function contacts; extracted threshold voltage values for (c) low work function contacts and (d) high work function contacts.

Supplementary Note 4: Degradation of silver contacts

The observation of decaying channel current in low work function devices suggests a high electro-chemical reactivity at the metal-semiconductor interface. **Figure S 7** shows optical images for the silver electrode devices after the measurement, where the deterioration of drain electrode after 6 long cycles manifests itself as bright optical contrast emerging at the edges of the positively biased drain electrode (**Figure S 7** (b). These observations suggest an electrochemical reaction taking place at the silver drain electrode interface during the S-D bias with prolonged bias resulting in severe degradation.

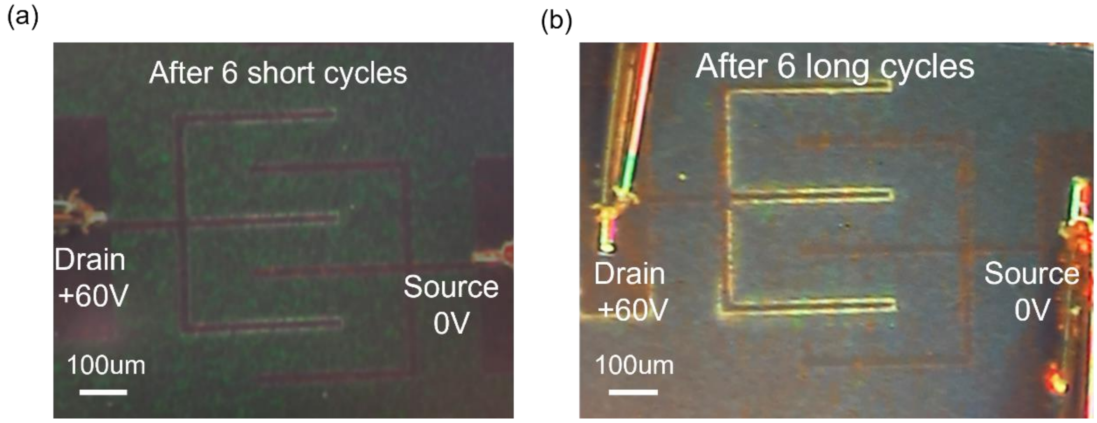

Figure S 7 Optical images of $Cs_{0.05}FA_{0.17}MA_{0.78}PbI_3$ (stoichiometric) FET devices with silver S-D contacts after six consecutive transfer curve measurements in (a) short cycle condition (s=4V, t=0.5s) and (b) long cycle condition (s=2V, t=1s). The devices in (a,b) are two different devices on a same chip. Measurement parameters: s=4V, t=0.5s. Channel length L=100µm, channel width W=1 mm. V_{DS}=+60V, V_{GS} is swept between -60V and +60V. Dielectric: 300nm SiO_2.

Supplementary Note 5: XPS measurements of electrode surfaces

XPS measurements were carried out to analyse the surface composition of the fresh electrodes prior to perovskite deposition. The fabrication method for XPS samples is described in Supplementary Note 2. **Figure S 8** shows Cr 2p spectra of Cr and CrO_x(1nm)-Cr films. The Cr $2p_{3/2}$ spectra in the region between 572 to 580 eV show a complex multiplet splitting. The peak at ~573.8 eV can be identified as metallic Cr. The wide peak at ~576 eV can be deconvoluted into multiple peaks associated with Cr(III) oxides and hydroxides. Comparing **Figure S 8** (a) to (b), the Cr spectrum shows a higher metallic Cr peak than the spectrum of CrO_x(1nm)-Cr. The data confirms the existence of Cr(III) oxide and hydroxide species on the as-fabricated Cr electrodes. The Cr oxide species could form in the process of lift-off and surface cleaning after thermal evaporation. Note that surface oxidation of chromium

electrodes is unavoidable and is difficult to control in the electrode lift-off process, thus the device performance from different batches of electrodes could slightly vary (**Figure 2** (e) compared to **Figure 3** (i)). The deposition of CrO_x onto Cr results in higher concentration of Cr(III) oxide species on the electrode surface.

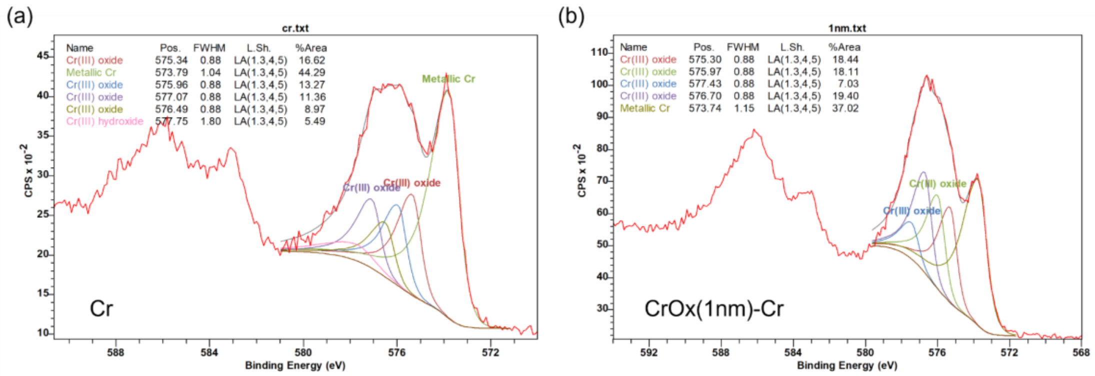

Figure S 8 XPS spectra of Chromium $2p_{3/2}$ peak of (a) as-deposited Cr metal (22nm) and (b) Cr-metal (22nm) modified with 1nm of CrO_x on top. Spectra were analysed by *CasaXPS* software.[12] A pass energy of 10 eV was used in the measurement.

The surface condition of Au, Pt and Ag electrodes was also analysed by XPS. **Figure S 9** shows the XPS spectra of Au 4f, Pt 4f and Ag 3d peaks for the respective metal electrodes. The spectra exhibit single peaks of Au $4f_{7/2}$ (Au $4f_{5/2}$), Pt $4f_{7/2}$ (Pt $4f_{5/2}$) and Ag $3d_{5/2}$ (Ag $3d_{3/2}$). No signature of metal oxide states was observed. In the silver spectrum, the small humps at 371.5 eV and 377.5 eV were due to plasmon losses.[13] The spectra suggest the as-fabricated noble metal electrodes are chemically stable prior to perovskite deposition.

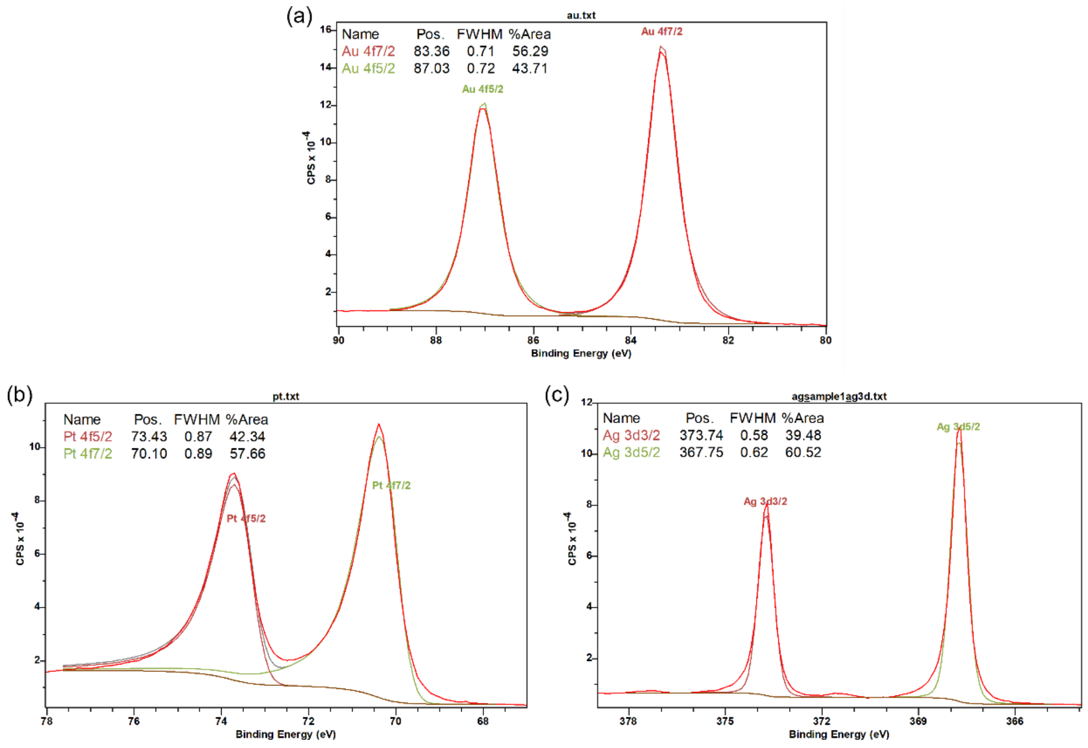

Figure S 9 XPS spectra of (a) Au 4f peak for gold electrode; (b) Pt 4f peak for platinum electrode; (c) Ag 3d peak for silver film. Spectra were analysed by *CasaXPS* software[12]. A pass energy of 20 eV was used in the measurement.

Supplementary Note 6: Channel current decay in devices with Cr and CrO_x electrodes

We further measured the transfer characteristics of $Cs_{0.05}FA_{0.17}MA_{0.78}PbI_3$ FETs with chromium source and drain electrodes modified by different CrO_x thickness. After the deposition of Cr in high vacuum ($2*10^{-6}$ mbar), the evaporation chamber pressure was increased to medium vacuum (10^{-3} mbar) by bleeding air into the chamber in order to deposit CrO_x. The thickness of CrO_x layer was estimated using a QCM crystal monitor using the same evaporation parameters (density, Z-ratio) as metallic Cr. The transfer characteristics of devices with different CrO_x electrode thicknesses are shown in **Figure S 10.** The extracted I_{D-max} upon repeated cycling in **Figure S 10** (d) shows that I_{D-max} dropped faster when the chromium oxide thickness increases. **Figure S 10** (e) shows that the electron mobility is also lower for devices with thicker CrO_x. The devices with approximately 5nm CrO_x exhibit an unusual S-shaped transfer characteristics which is reflected also in anomalously high threshold voltages extracted for these devices (**Figure S 10** (f)). After cycling, there is a pronounced upshift in the threshold voltage in CrO_x devices, suggesting p-doping process taken place during the bias. These results suggest CrO_x source and drain electrodes degrade electron injection and results in instabilities under prolonged channel bias. As **Figure S 8**

proves the presence of oxide states of Cr on the electrode surface, the instability of chromium oxide species could be partially responsible for the current decay in Cr electrode devices.

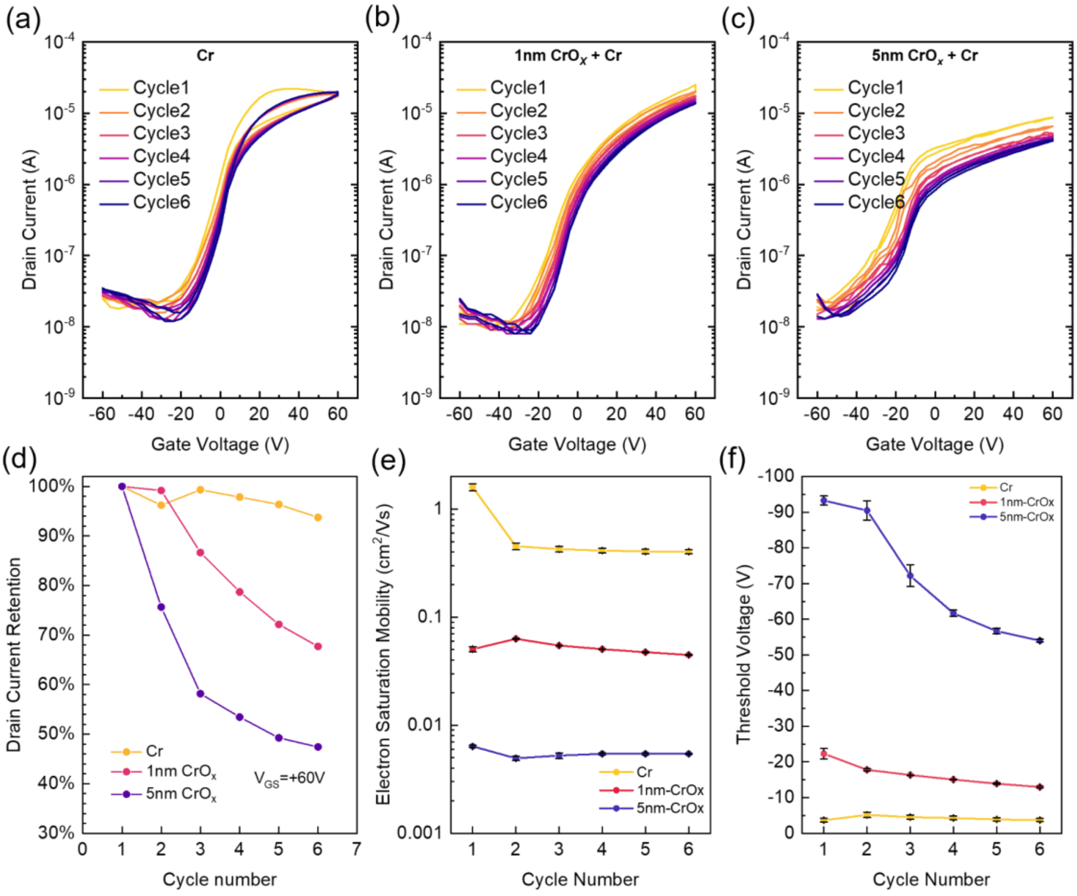

Figure S 10 Transfer characteristics of 6 consecutive measurement cycles of $Cs_{0.05}FA_{0.17}MA_{0.78}PbI_3$ FET devices with chromium S/D electrodes with different CrO_x modification layer thickness: (a) Cr (22nm) without CrO_x; (b) Cr (22nm) with 1nm CrO_x; (c) Cr (22nm) without 5nm of CrO_x. (d) $I_{D\text{-}max}$ retention change of different samples at each measurement cycle, calculated by $I_{D\text{-}max}$ (n^{th} cycle):$I_{D\text{-}max}$ (1st cycle) *100%; (e) Extracted saturation electron FET mobility and (f) threshold voltage. Measurement parameters: s=4V, t=0.5s. Channel length L=100μm, channel width W=1 mm. V_{DS}=+60V, V_{GS} sweeps between -60V and +60V. Dielectric: 300nm SiO_2.

Supplementary Note 7: Detailed transport characterization on samples with different stoichiometry under S-D bias

$\mu_{e,FET}$ values of each sample in **Figure 3** are extracted from their saturation regime transfer curves by the following equation:

$$\mu_{e,FET} = \frac{2L}{W \cdot \varepsilon_0 * \varepsilon_r / \tau} \cdot \left(\frac{\partial\sqrt{|I_D|}}{\partial V_{GS}}\right)^2$$

L: channel length; W: channel width; ε_0: vacuum permittivity; ε_r: relative permittivity of the dielectrics; τ: dielectric layer thickness.

The variation of $\mu_{e,FET}$ with sample stoichiometry and cycle number is shown in **Figure S 11**, where $\mu_{e,FET}$ follows similar changing trend as $I_{D\text{-}max}$.

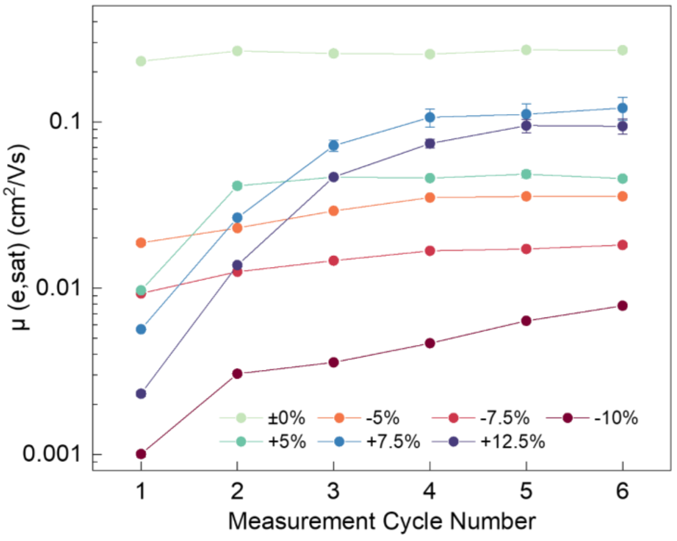

Figure S 11 $\mu_{e,FET}$ extracted from Figure3 for $Cs_{0.05}FA_{0.17}MA_{0.78}PbI_3$ in different compositions.

After the six measurement cycles, we rested the samples for a certain time and measured them again. All the samples were rested for 20 min after a first set of measurement cycles and some samples were further rested for 25 min after a second set of measurement cycles. After resting, 3-4 transfer characteristics measurements were carried out consecutively. The I_D at V_G=+60 V of these measurements are shown in **Figure S 12**. All the samples experienced a drop in I_D after rest and an increase in I_D after repeated cycling except the stoichiometric one (±0%). This shows that the current build-up effect is reversible. In some samples, there is an increase of $I_{D\text{-}max}$ after the rest.

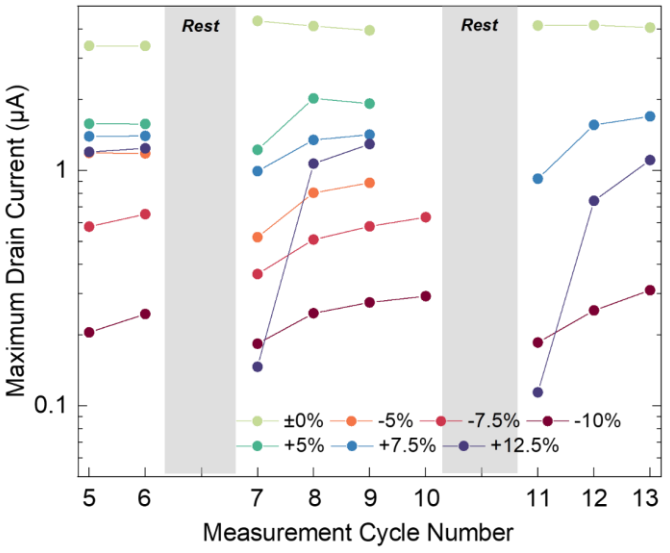

Figure S 12 $I_{D\text{-}max}$ change after repeated measurement cycles with rest periods in between. Devices were rested for 20 minutes between cycle 6 and 7, then 25 minutes between cycle 10 and 11. The same short-cycle parameters (t=0.5s, s=4V) were applied for measurements after cycle 6.

The absolute values of $I_{D\text{-}max}$ of three typical samples from **Figure 3** (d) are compared in **Figure S 13**. PbI_2-stoichiometric sample exhibited the highest ON current before and after bias. The PbI_2-excess device showed the highest ON current increase of 11.8 µA after 6 cycles. The PbI_2-deficient device exhibited the smallest absolute increase of 2.9 µA, but a relatively high increase ratio of 250% as shown in **Figure 3** (h). The lowest ON current in PbI_2-deficent sample could mostly be due to the background p-doping effect caused by the excess MAI/FAI/CsI.

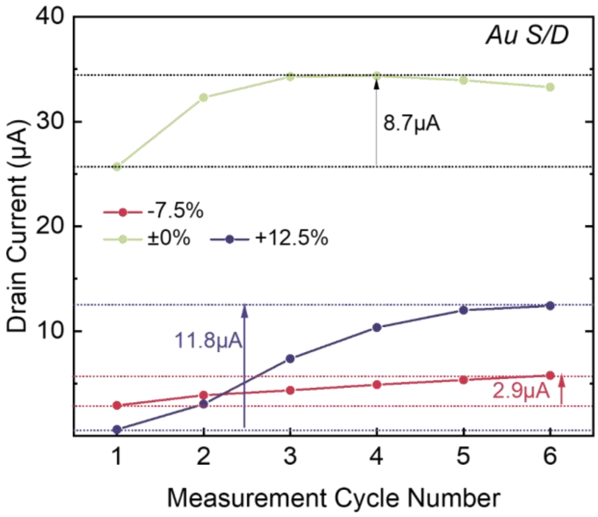

Figure S 13 $I_{D\text{-}max}$ change after repeated measurement cycles in stoichiometric, -7.5mol% PbI2 deficient and +12.5mol% PbI2 excess devices with y-axis in linear scale.

To evaluate longer time operational device stability, we further measured the $Cs_{0.05}FA_{0.17}MA_{0.78}PbI_3$ devices (with Au electrodes) in stoichiometric, PbI_2-deficient, and PbI_2-excess compositions for up to 70 transfer cycles. The first 9 cycle transfer curves are shown in **Figure S 14** (a)-(c). After cycle 9, devices were rested for 1 minutes before commencing the rest 61 cycles. The $I_{D\text{-}max}$ values for later cycles were plotted in **Figure S 14** (d). The non-stoichiometric samples exhibited a clear current build-up effect as expected, though the current increase ratio is not as pronounced as in **Figure 3**, and the initial $I_{D\text{-}max}$ of these samples is also higher. These differences can be attributed to variations in sample preparation conditions across different batches. As can be also seen in **Figure S 12**, there was an increase of $I_{D\text{-}max}$ after the 1-minute rest at cycle 11 and 12. Upon further cycling, all the samples experienced a decay of $I_{D\text{-}max}$ in a similar pattern. $I_{D\text{-}max}$ in non-stoichiometric samples decayed fast between cycle 12 to cycle 30 and then dropped at a smaller rate until cycle 70. $I_{D\text{-}max}$ in stoichiometric sample dropped at a steady rate from cycle 12 to cycle 70. This long-term operational degradation is an important phenomenon, which looks similar to the current degradation reported on two terminal perovskite devices subjected to long biasing time. [14,15]. In this work we focus on the understanding of current change for devices subjected to shorter time scales of bias-stress, which exhibits a distinctive dependence on sample stoichiometry.

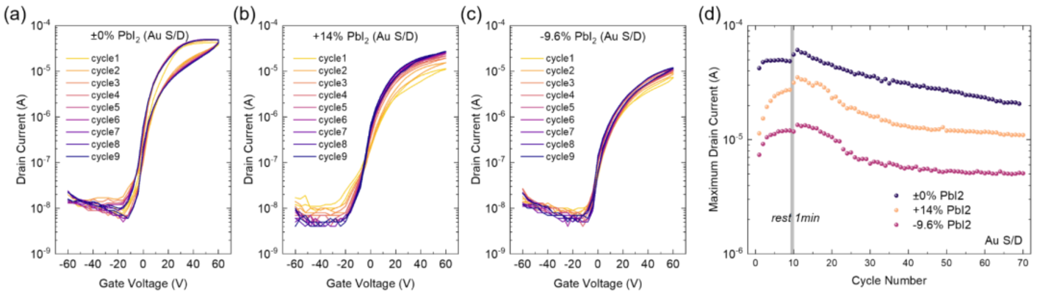

Figure S 14 Long time stability tests for stoichiometric and non-stoichiometric $Cs_{0.05}FA_{0.17}MA_{0.78}PbI_3$ perovskite FETs with gold source and drain electrodes. First 10 cycles' transfer characteristics for devices with (a) stoichiometric, (b) +14 mol% excess PbI_2 and (c) -9.6 mol% deficient PbI_2 compositions. (d) Evolution of maximum drain current (I_{D-max}) of the above devices in 70 cycles. Measurement parameters: s=4V, t=0.5s. Channel length L=100µm, channel width W=1 mm. V_{DS}=+60V, V_{GS} sweeps between -60V and +60V. Source and drain electrodes: 22 nm Au. Dielectric: 300nm SiO_2.

We further carried out temperature-dependent transfer characteristics measurement on stoichiometric, PbI_2-excess, and PbI_2-deficient samples at 300K, 250K, 150K, and 100K and extracted the saturation FET mobility values, as shown in the following figure. FET devices (L=100um, W=1mm) are measured for six consecutive cycles at each temperature. The stoichiometric device shows higher mobility than PbI_2-excess/deficient devices at low temperatures (250, 200K, 150K). Overall, at low temperature the mobility increase was less pronounced than at room temperature (300K). The mobility for stoichiometric (from 0.0017 cm^2/Vs to 0.002 cm^2/Vs, 1.18 times) and PbI_2-deficient (5.2E-4 cm^2/Vs to 6.5E-5 cm^2/Vs, 1.25 times) devices barely changed at 150K. The PbI_2-excess device, compared with itself, exhibited the greatest mobility increase (from 0.0026cm^2/Vs to 0.4 cm^2/Vs, 150 times) at 300K and the lowest (from 0.0052cm^2/Vs to 0.02 cm^2/Vs, 3.8 times) at 150K. The PbI_2-deficient sample showed the greatest mobility increase at 250K (from 0.03cm^2/Vs to 0.12 cm^2/Vs, 4 times) and the lowest at 150K (5.2E-4 cm^2/Vs to 6.5E-5 cm^2/Vs, 1.25 times). The temperature-dependent measurement shows that the repeat-cycle induced mobility increase is only pronounced at high temperatures and is suppressed at low temperatures. The large mobility increases in the PbI_2-excess sample compared to the small mobility increase in the PbI_2-deficient sample suggests the defect landscape is different in these two compositions, where defects in the PbI_2-excess sample seem to be more mobile at both high and low temperatures. This result could be correlated with the fast electromigration of Au in PbI_2-excess environments indicated in **Figure 6** in the main paper.

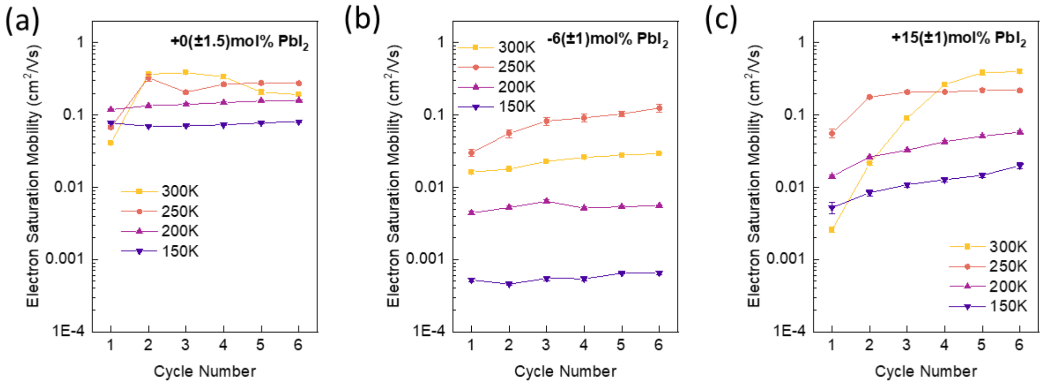

Figure S 15 Extracted $\mu_{e,FET}$ for six consecutive cycles measured at different temperatures for Au $Cs_{0.05}FA_{0.17}MA_{0.78}PbI_3$ in different compositions. (a) Stoichiometric sample (composition error: ±1.5 mol% PbI_2); (b) PbI_2 deficient sample with -6(±1) mol% PbI_2; (c) PbI_2 excess sample with +15(±1) mol% PbI_2. Measurement parameters: s=4V, t=0.5s. Channel length L=100μm, channel width W=1 mm. V_{DS}=+60V, V_{GS} sweeps between -60V and +60V. Dielectric: 300nm SiO_2.

Supplementary Note 8: Observation of weak p-type transport in top-gated device

While all our bottom-gate devices were n-type we observed weakly p-type transport in PbI_2-deficient samples in top-gate, bottom-contact FETs. **Figure S 16** shows the transfer characteristics of the weakly p-type devices with different PbI_2 deficiency ratio. The inserted schematics in **Figure S 16** (a) shows the structure of a top-gate, bottom-contact FET. The I_{D-max} of these devices is lower than 20 nA, and the highest on-off ratio is also lower than 50. In principle, p-type operation is expected when using PbI_2-deficient perovskite precursors. Paul et al.[16], and Wang et al.[17] already observed the shift from n-type to p-type doping upon changing the $MAPbI_3$ or $FAPbI_3$ precursor stoichiometry from PbI_2-excess to PbI_2-deficient. Nevertheless, PbI_2-deficient devices in bottom gate, bottom contact structure exhibit clear n-type transport in **Figure 3** (e)-(g). The reason for this dependence on device geometry is not clear at present. It could be related to differences in defect formation between the top surface of the perovskite film and the buried perovskite-substrate interface or it could be due to differences in the electronic structure of the perovskite-SiO_2 and perovskite-Cytop interfaces.

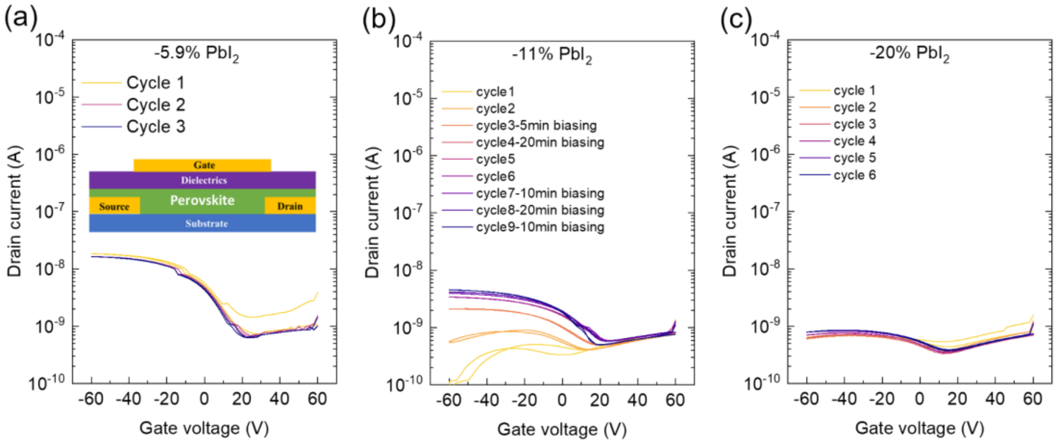

Figure S 16 Transfer characteristics of repeated measurement cycles for p-type $Cs_{0.05}FA_{0.17}MA_{0.78}PbI_3$ (PbI_2-deficient) FETs with different compositions: (a) -5.9 mol% deficient PbI_2; (b) -11 mol% deficient PbI_2, the device was biased at V_{GS}=V_{DS}=-60V for multiple times; (c) -20 mol% PbI_2. Note the device was in a top gate, bottom contact (TG, BC) geometry, with 500 nm Cytop as dielectrics and 20 nm Au as top gate. The inserted diagram in (a) shows the cross-section of the TG, BC architecture. Transfer curves were measured using long-cycle measurement condition with (s=2V, t=1s). Channel length L=100μm, channel width W=1 mm. V_{DS}=+60V, V_{GS} is swept between -60V and +60V. Source and drain electrodes: 22 nm Au.

Supplementary Note 9: Detailed XRD characterization of stoichiometric and non-stoichiometric $Cs_{0.05}FA_{0.17}MA_{0.78}PbI_3$ films

Detailed XRD curves were measured with a small 2θ step to investigate any shifts of the perovskite (110) peak at 13.95°. The spectra are shown in **Figure S 17**. PbI_2 excess samples show the highest PbI_2 (001) peak at 12.6° and perovskite (110) peak at 13.95°. The high peak intensity of perovskite (110) peak in PbI_2 excess films is also reported by Liao et al.[18]. The improved crystallinity of perovskite film could be attributed to the passivation effect of excess PbI_2 [19]. **Figure S 17** (b) shows normalized plots of the perovskite (110) peak. The peak position in the non-stoichiometric samples exhibits a small shift of 0.02° to smaller scattering angles for both PbI_2 excess and deficient conditions when compared to the stoichiometric films. This lattice expansion could be attributed to a lattice expansion upon incorporation of defect species[20] under non-stoichiometric conditions.

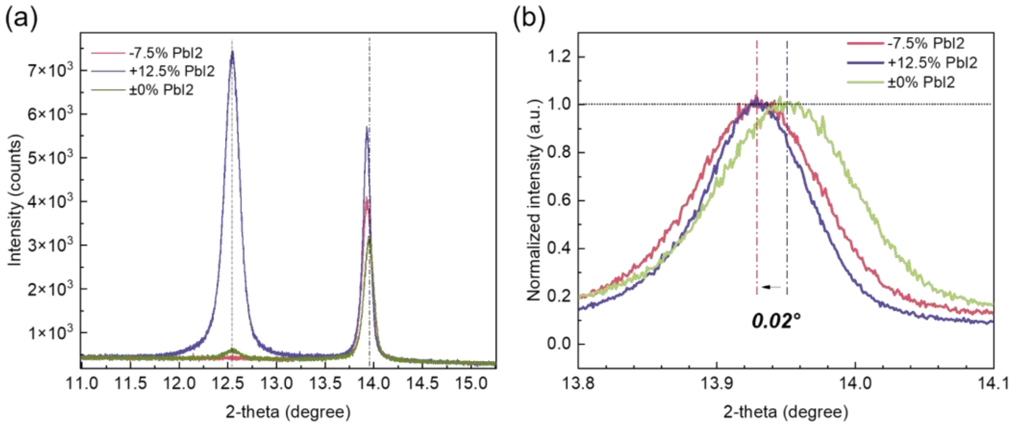

Figure S 17 Detailed XRD curves of stoichiometric, PbI_2-excess and PbI_2-deficient $Cs_{0.05}FA_{0.17}MA_{0.78}PbI_3$ perovskite films plotted in (a) on the as measured intensity scale and (b) on a normalized intensity scale. Scans were carried out with 2θ step of 0.001° and scan rate of 4 minutes per degree. The curves were calibrated using Au (111) peak position at 38.131°.

Supplementary Note 10: Photoluminescence mapping analysis on biased Au and Cr devices with different stoichiometry

A detailed analysis of PL mapping spectra is shown in **Figure S 18**. The Y-axis scales are optimised for each device to exhibit the detail of the spectra. **Figure S 18** (e,f) indicate that the PbI_2-deficient films have the weakest PL signal intensity among the three composition samples, regardless of the electrode material. In **Figure S 18** (c,d) for devices after bias stress (ii and iii), PbI_2-excess films maintain a high PL intensity in the centre of the channel area (x=20 μm) among the three compositions, which is even greater than that of PbI_2-stoichiometric films (**Figure S 18** (a,b)). The stoichiometry dependence of PL signal intensity could be correlated with the quantity of native iodine interstitials I_i^- in the film. High density of iodine interstitials in MAI-rich film[21] can effectively suppress the PL signal from the film. An excess of PbI_2 can also eliminate the unreacted MAI/FAI and reduce I_i^- density, contributing to the high and stable PL in these films.

All the films exhibited a PL quench near the positively biased electrode. The PL quenching was more pronounced for the Au devices (**Figure S 18** (a,c,e) vs. (b,d,f)), suggesting the PL quench is related to the electrode material used. Cr devices (PbI_2-stoichiometric and excess) showed a high PL signal intensity on the Cr electrodes (**Figure S 18** (b,d)), compared to the complete PL quenching on Au electrodes (**Figure S 18** (a,c)). These observations suggest the PL quenching in Au devices could be partially resulted from the electromigration of Au ions from the electrodes. The wide PL quench in PbI2-excess (Au) film could also be related to the

faster electromigration of Au ions at the presence of excess PbI_2, as supported by the evidence from **Figure 6** (b,f) in the main paper. There was a noticeable PL brightening in the middle part of the channel in PbI_2-deficient (Cr and Au) and PbI_2-stoichiometric (Cr) films, as marked by the up arrows in **Figure S 18** (b,e,f). The PL brightening could be explained by the compensation effect of V_I^+ on I_i^- as suggested by Li et al.[14]

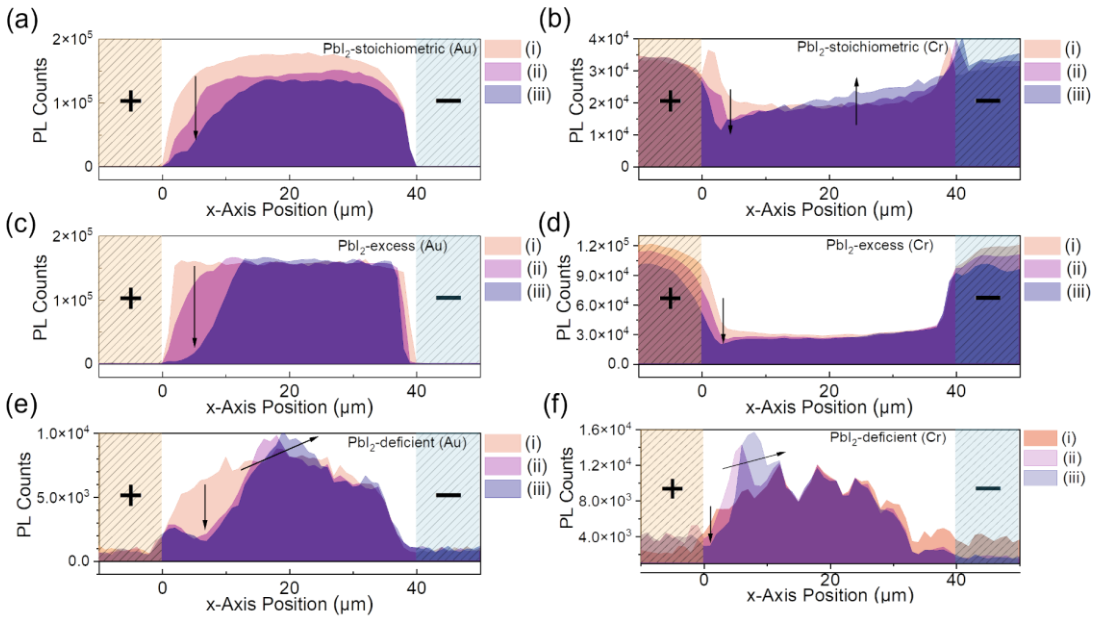

Figure S 18 Detailed Y-axis averaged PL intensity profiles plotted in linear scale correspond to the bottom panels in Figure5. The Y-scales are properly adjusted for each sample to fully exhibit PL changes during biasing. The rise and fall of PL intensity in PL quenching and brightening regions are marked by arrows in each graph, respectively.

To investigate the recovery of the changes in the PL maps observed **in Figure 5** the stoichiometric device with gold electrodes was rested in the dark for 8 minutes and imaged again. The PL images and profiles before and after rest are shown in **Figure S 19**. After the rest, there was a PL recovery in the channel centre (PL intensity increased +15% at x=20 μm) and near the originally quenched area (PL intensity increased +49% at x=3.9 μm). This suggests that the bias-induced PL quenching effect near the gold drain electrode devices is at least partially reversible.

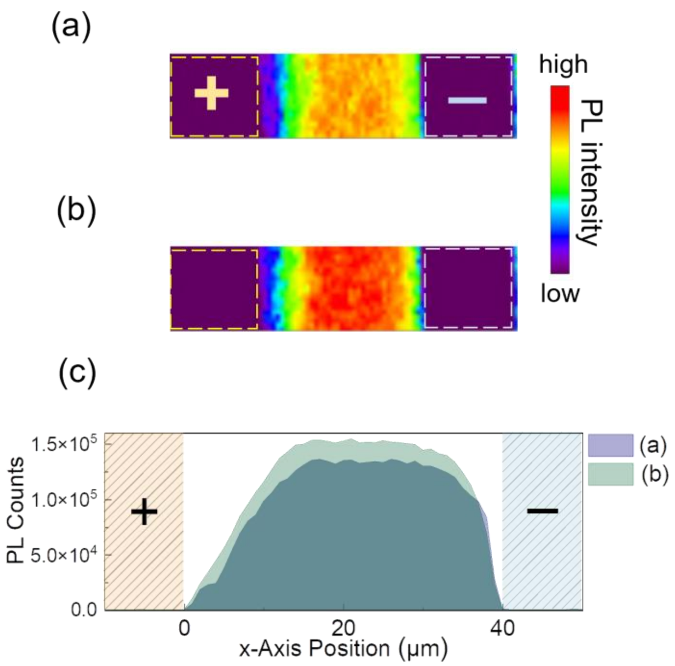

Figure S 19 PL images and profiles of a stoichiometric $Cs_{0.05}FA_{0.17}MA_{0.78}PbI_3$ samples with gold electrodes before and after resting 8 minutes in dark. (a) PL images taken immediately after twice application of a (30s, 30V) bias, which is showing the same image as the third stage in Figure5(a); (b) PL images taken after resting the device in dark for 8 minutes; (c) Y-axis averaged PL intensity profiles for the above two stages. Channel length L=40μm, channel width W=1 mm.

In **Figure 5** (c) PbI_2-deficent (Au) film, we notice that there is a hump in the PL profile close to the positive electrode after biasing. **Figure S 20** shows the PL maps at other locations on the same film, where no hump is observed along the positive electrode edge. This suggests the hump in **Figure 5** (c) is most likely due to some artefacts.

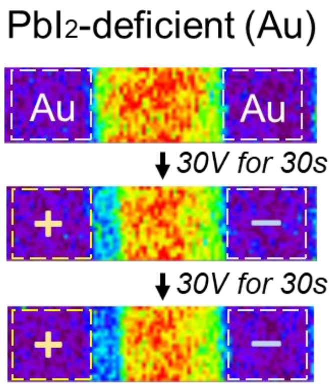

Figure S 20 Photoluminescence response of PbI_2-deficient $Cs_{0.05}FA_{0.17}MA_{0.78}PbI_3$ perovskite with Au contacts under a DC bias. The images are taken from a different area on the same device as Figure 6 (c). The color scale is the same as Figure 6 (c).

Supplementary Note 11: Ex situ SEM and EDX analysis on biased Au devices with different stoichiometry

The current build-up effect and PL quench could be the result of bias-stress induced electrochemical reactions at electrode-perovskite interfaces. These interfaces were further analysed by SEM and EDX. **Figure S 21** shows the morphology of perovskites at Au electrode edges in a bottom gate, bottom contact FET after moderate bias (10 transfer sweeps). The Au particles were visible near the Au drain edge, which is consistent with the results shown in **Figure6** (a)-(c) for overly bias-stressed devices. Here the moderately biased device showed less Au particles compared to the overly biased devices.

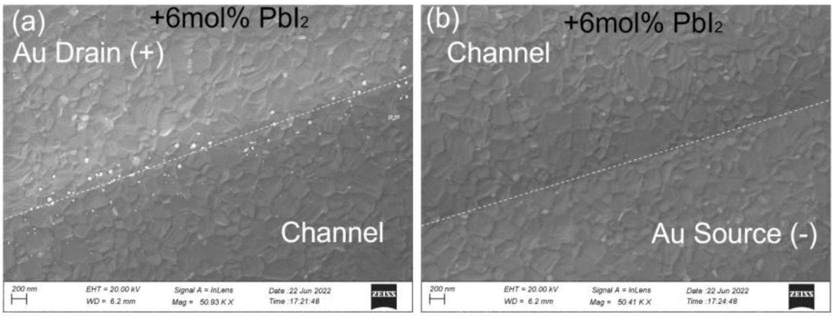

Figure S 21 SEM images of PbI_2-excess (+6mol% PbI_2) $Cs_{0.05}FA_{0.17}MA_{0.78}PbI_3$ perovskite FET after moderate bias (10 transfer sweeps) showing Au nanoparticles at Au drain fringe in (a) and no features at (b) Au source fringe.

The perovskite layer was subsequently removed to investigate the bias-induced degradation of the Au electrodes. **Figure S 22** shows the SEM morphology of the Au source electrodes corresponding to devices showed in **Figure 6** (e)-(g). **Figure S 23** shows the morphology of the entire electrodes bar for biased and unbiased devices at a low SEM magnification. The bars have a width of 20 µm and length of 500 µm. The source and drain bars are finger interdigitated therefore both edges of the bar were bias-stressed and showed a symmetrical morphology.

The degradation of drain electrode edge is evident in **Figure S 23** (g,h,i) with the most severe degradation observed in the PbI_2-excess composition. There was no noticeable Au cluster formation at the source electrode edges in all compositions, but the electrode surfaces near the edge appeared darker than the interior in PbI_2-stoichiometric and excess devices. The width of the dark belt is 1.5 µm for PbI_2-excess device and 0.9 µm for PbI_2-stochimetric device. The

Au grain size appeared to be larger in PbI_2-deficient devices (**Figure S 22** (c)) and was even discernable under low SEM magnification images (**Figure S 23** (c,f,i)**).**

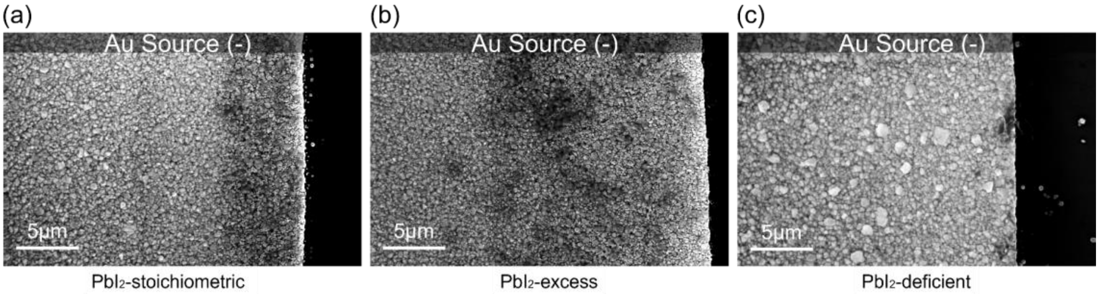

Figure S 22 SEM images of the exposed Au source electrodes for (a) stoichiometric, (b) PbI2-excess, and (c) PbI2-deficient films. These electrodes are from the same devices corresponds to Figure6e, f, g, which are biased for 150 transfer sweeps. Here, (a) and (b) show the surface near the Au source edge appears darker.

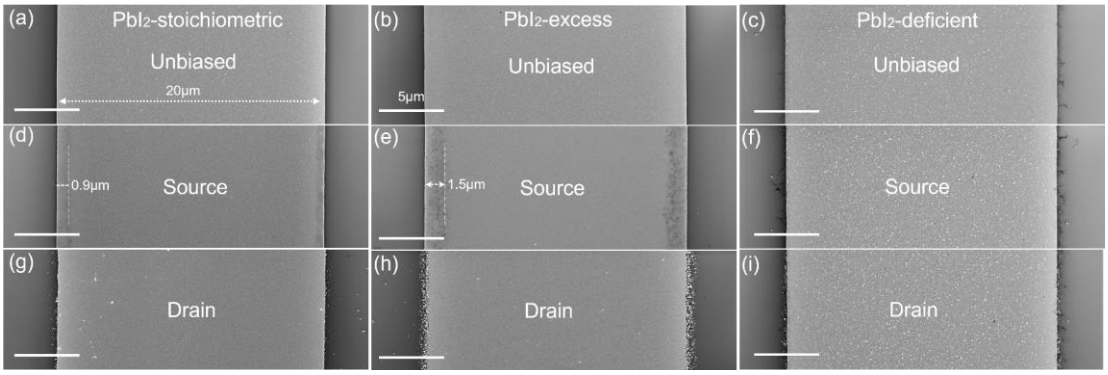

Figure S 23 SEM images of the exposed Au electrodes for (a) stoichiometric, unbiased, (d) stoichiometric, source, (g) stoichiometric, drain, (b) PbI_2-excess, unbiased, (e) PbI2-excess, source, (h) PbI_2-excess, drain, (c) PbI2-deficient, unbiased, (f) PbI_2-deficient, source and (i) PbI_2-deficient, drain. These electrodes are from the same devices correspond to Figure 6 (d,e,f), which are biased for 150 transfer sweeps. All SEM images have a scale bar of 5 µm.

EDX elemental mapping was also performed on the Au edge regions for source (**Figure S 24**) and drain (**Figure S 25**) electrodes. There was no noticeable redistribution of Au, Pb and I at source electrode edges for all compositions. As discussed in the main paper, the dark belt on the source electrode could be due to the aggregation of iodine species. There was no contrast difference between the electrode and channel regions in the iodine maps, which suggests that the trace amount change in iodine species here might be too small to be detected by the EDX method.

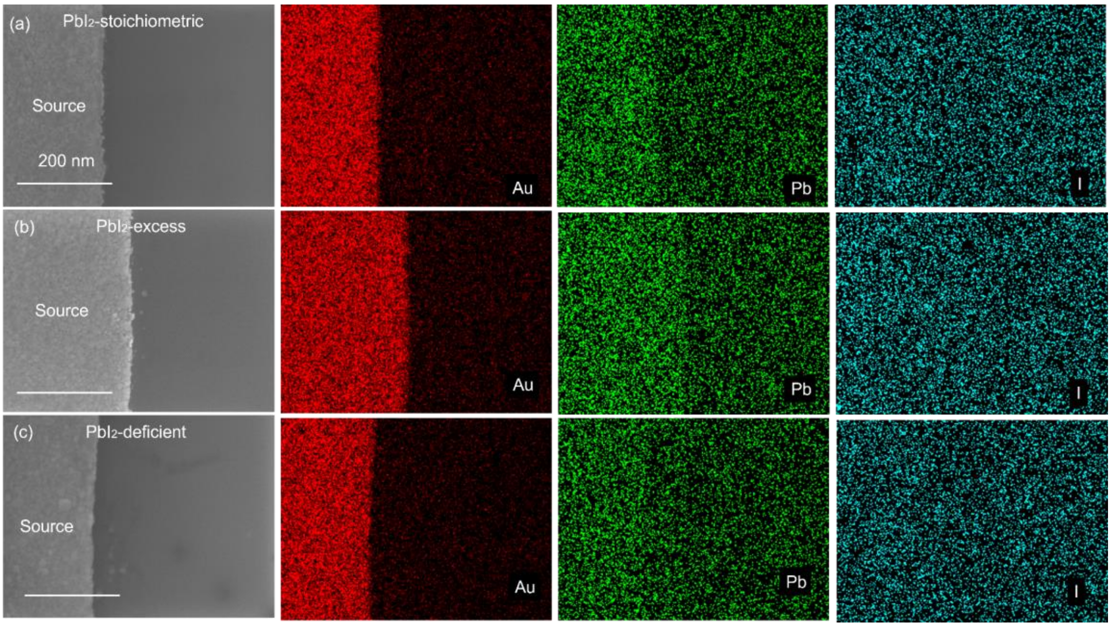

Figure S 24 SEM and EDX mapping images of the exposed Au source electrodes for (a) stoichiometric, (b) PbI_2-excess and (c) PbI_2-deficient samples. These electrodes are from the same devices correspond to Figure6 (d, e, f), which are biased for 150 transfer sweeps. All images have a scale bar of 200nm. The small particles near the electrodes may be residual from the DMF spin-coating removal process.

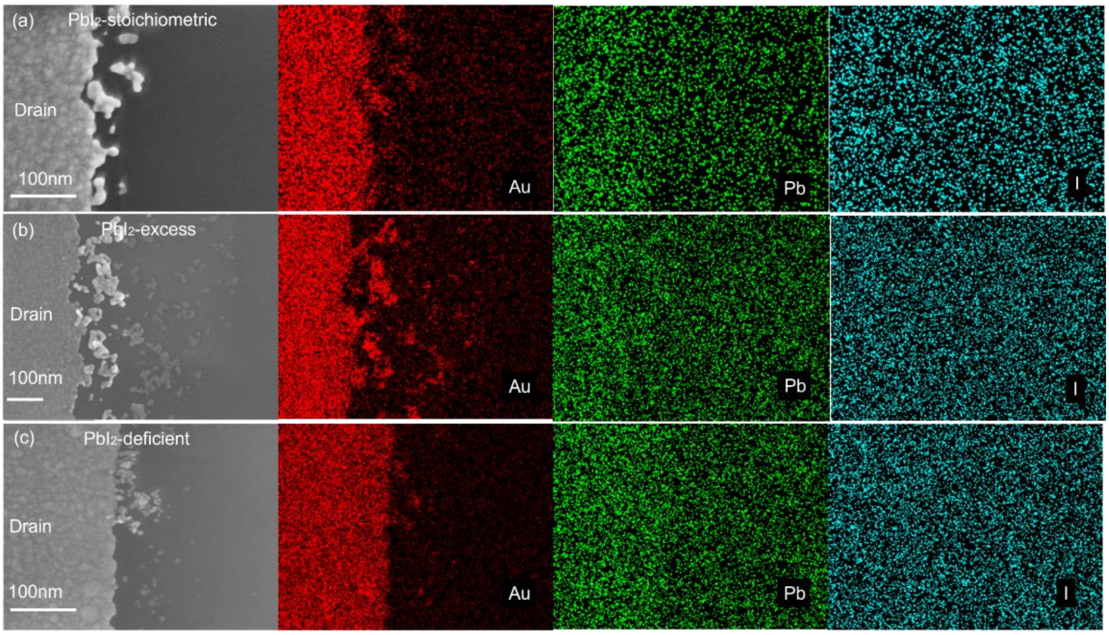

Figure S 25 SEM and EDX mapping images of the exposed Au drain electrodes for (a) stoichiometric, (b) PbI_2-excess and (c) PbI_2-deficient samples. These electrodes are from the same devices correspond to Figure6d, e, f, which are biased for 150 transfer sweeps. All images have a scale bar of 100nm.

Supplementary Note 12: SEM and EDX analysis of a pure-PbI_2 FET device after long term bias stress

The discussion in the main paper suggests the presence of PbI_2 grains speeds up the electromigration of Au from positively biased drain electrode. To verify this, SEM and EDX analysis were conducted on a bias-stressed pure-PbI_2 FET device. We used a channel length of 5 µm to better exhibit the changes from source to drain. **Figure S 26** shows a significant amount of Au particles formed on top of the PbI_2 grains and extended from the positive drain to the vicinity of the source. This result supports the theory that the presence of PbI_2 grains accelerates the electromigration of Au.

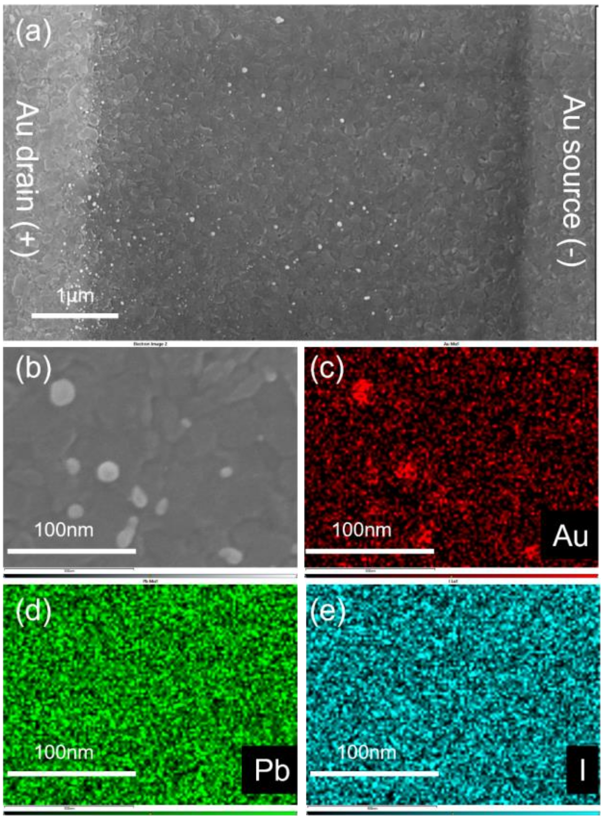

Figure S 26 SEM and EDX mapping images of the channel and electrodes for a pure PbI_2 film after long term bias stress testing (100 transfer sweeps at V_{DS}=+60V and V_G from 0 to +60V). The Au particles are visible from the Au drain to the channel. Channel length is 5um.

Supplementary Note 13: Ex situ SEM and EDX analysis on biased Cr devices

SEM and EDX mapping analysis were performed on a device with Cr electrodes after bias stress. Unlike the Au devices, no particle were found on top of the perovskite layer near the electrodes in **Figure S 27**. However, after removing the perovskite layer, bright particles were observed at the Cr source edge in **Figure S 28** (c). EDX mapping revealed that these particles were made of Pb (**Figure 6** (h)). No Cr or Pb particles were found near the drain electrode

edge (**Figure S 29**). These results suggest that in the case of Cr electrode the reduction of Pb^{2+} to Pb occurs at the negatively biased electrode.

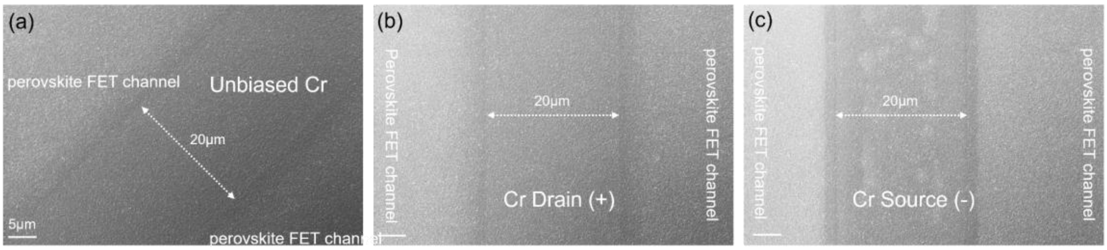

Figure S 27 SEM images of the Cr electrode area in a stoichiometric $Cs_{0.05}FA_{0.17}MA_{0.78}PbI_3$ perovskite FET for (a) unbiased, (b) Cr drain and (c) Cr source. Electrode width is 20um. The device in (b) and (c) is biased for 200 transfer sweeps. All images have a scale bar of 5um. Images are taken before the removal of perovskite layer.

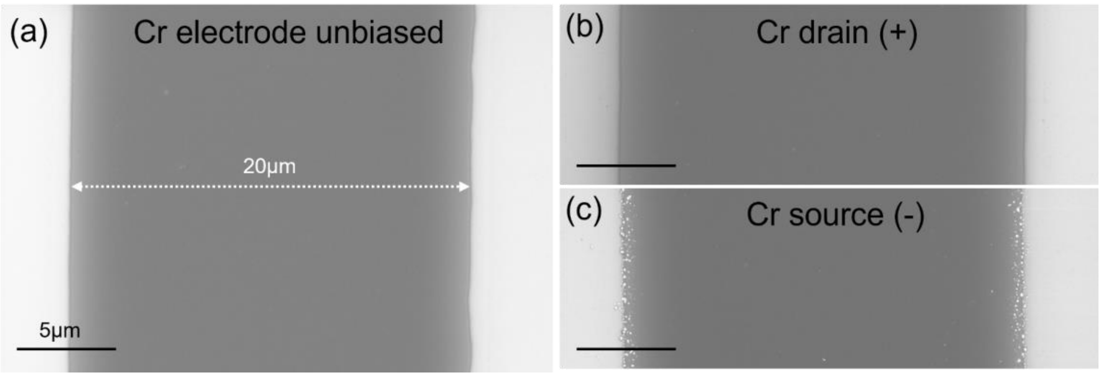

Figure S 28 SEM images of the exposed Cr electrode areas in a stoichiometric $Cs_{0.05}FA_{0.17}MA_{0.78}PbI_3$ perovskite FET for (a) unbiased, (b) Cr drain and (c) Cr source. Electrode width is 20 μm. All images have a scale bar of 5um. Images are taken after the removal of perovskite layer. Bright Pb particles are visible at Cr source electrode fringe. These electrodes are from the same sample corresponds to Figure 6 (h), which is biased for 200 transfer sweeps.

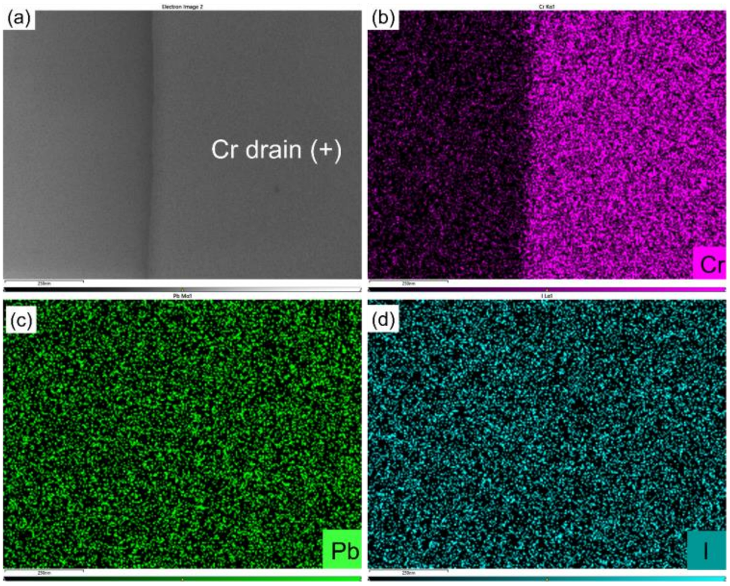

Figure S 29 EDX mapping of the Cr drain electrode fringe in a stoichiometric $Cs_{0.05}FA_{0.17}MA_{0.78}PbI_3$ perovskite FET after removing the perovskite layer. The drain electrode shown here is from the same device corresponds to Figure 6 (h), which was biased for 200 transfer sweeps.

Supplementary Note 14: Diagrams illustrating the potential changes in the band structure following interfacial n-doping

Perovskite FETs exhibit different types of anomalous transport behaviors depending on the work function of contact metal and sample stoichiometry. To aid the discussion of mechanisms in the main paper, we present schematic diagrams here to show the change in band diagram in detail. **Figure S 30** (a) schematically shows the relative positions of the metal work function and the bandgap of an n-type perovskite semiconductor. For high work function materials, the large energy difference between the electrode work function and the perovskite Fermi level (E_F) results in a bending of the conduction band and formation of a high Schottky barrier for electron injection (**Figure S 30** (b)). Without the gate field applied, electrons need to overcome the barrier by thermal activation (thermionic emission)[22]. When applying a S-D bias, some ion migration processes lead to the n-doping of the interfacial region. The thinning of the depletion zone at the source-semiconductor interface facilitates electron tunneling through the energy barrier (**Figure S 30** (c)). For low work function metals, thermionic emission of electrons is easier due to the low Schottky barrier height (**Figure S 30** (d)). However, owing to the high chemical reactivity of low work function metals, the perovskite might react with the electrode more readily leading to the formation of deep electron traps at the interfaces or even to the formation of electrically insulating interfacial barrier layers.

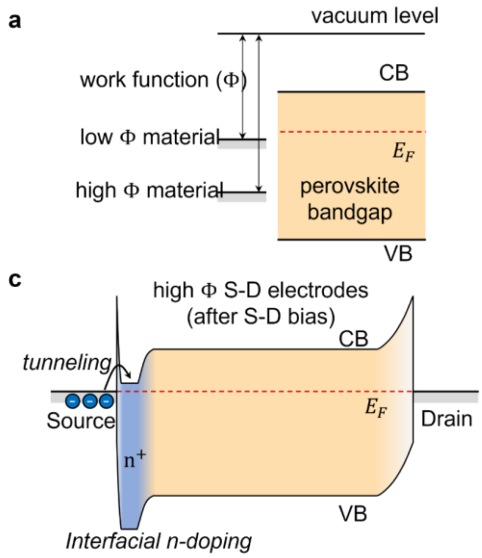

Figure S 30 The effect of source and drain work function and defect migration on the charge transport of a n-type lead triiodide perovskite FET. (a) Schematics of the relative energy positions between perovskite bandgap and metal work function (Φ). (b) Energy diagram for a perovskite FET channel with high Φ electrodes, where a high Schottky barrier is formed at the metal-semiconductor (M-S) interface. Electron injection is mainly thermionic when V_{GS}=0V. (c) Energy diagram after the S-D bias. Ion migration processes create an n+ region at the source side M-S interface, which reduces the depletion zone thickness. Electron injection can take place via tunnelling through the thin energy barrier. (d) Energy diagram of a perovskite FET channel with low Φ electrodes. A low Schottky barrier is formed with negligible band bending. Though electron injection is easier, the electrode-perovskite reaction creates some deep electron traps at M-S interfaces.

Supplementary Note 15: The effect of Au^+ doping

Previous measurements suggest the current build-up effect could most likely due to a process involving the n-type doping effect of Au^+ ions migrated from the positive biased drain electrode. To confirm this we introduce Au^+ into the film by doping the perovskite precursor with trace amount of gold monoiodide (AuI) with a molar ratio of 0.5 mol% to 2 mol% and carry out repeated transfer measurement sweeps (long-cycle) to the devices. The base perovskite composition used here is stoichiometric $Cs_{0.05}FA_{0.17}MA_{0.78}PbI_3$. The transfer characteristics of 7 consecutive sweeps are shown in **Figure S 31**. The pristine device exhibits a small increase ON current from 30 µA (cycle 1) to 32 µA (cycle 2) by 6% and then a steady drop to 21 µA until cycle 7. The 0.5mol% AuI doped device show a steady increase in ON current from 10 µA (cycle 1) to 20 µA (cycle 4) by 200% and a slight drop to 16 µA until cycle 7. The 2mol% AuI doped device shows a slow increase in ON current from 1 µA (cycle 1) to 4 µA (cycle 7) by 400%.

The influence of external I^- from AuI could be negligible as a doping ratio of +5mol% I^- (-5mol%

PbI_2 sample in **Figure 1** (e)) did not result in a significant change of ON current comparable to the +2 mol% I^- device showed in **Figure S 31** (c). Then the drastic change of initial ON current and pronounced current build-up effect there could be attributed to the external Au^+ ions. The long and significant current increase due to Au^+ doping is possibly realized by a process involving the lateral migration of doped Au^+ to the electrode interface and improved electron injection due to interfacial n-type doping. In normal situations without AuI doping, Au^+ comes from the oxidation and electromigration of the positively biased Au drain electrode, as indicated in **Figure 6** from the main paper.

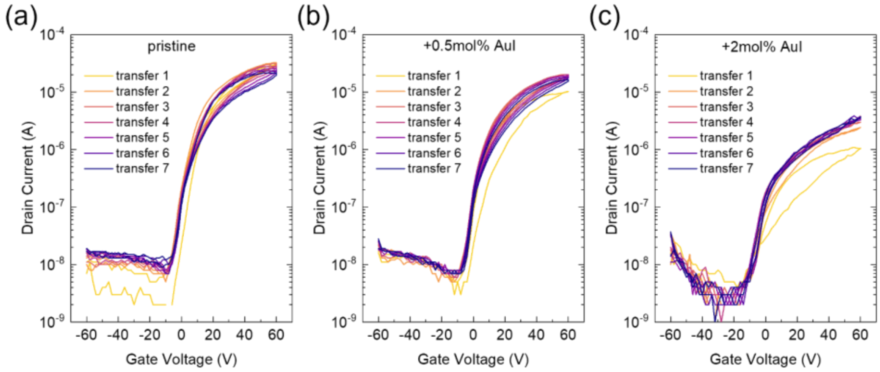

Figure S 31 Transfer characteristics of gold monoiodide (AuI) doped $Cs_{0.05}FA_{0.17}MA_{0.78}PbI_3$ perovskite FETs for 7 consecutive measurement cycles. (a) undoped pristine sample, (b) doped by 0.5 mol% of AuI, (c) doped by 2 mol% of AuI. Transfer characteristics are measured in long cycles (s=2V, t=1s). The AuI doped samples exhibits increase in ON current upon cycling, compared to the pristine sample which did not show any increase.

References:

[1] A. J. Szadkowski, A. Kalnitsky, K. B. Ma, S. Zukotynski, *J. Appl. Phys.* **1982**, *53*, 557.
[2] J. R. Waldrop, *J. Vac. Sci. Technol. B Microelectron. Process. Phenom.* **1984**, *2*, 445.
[3] H. B. Michaelson, *J. Appl. Phys.* **1977**, *48*, 4729.
[4] J. Hölzl, F. K. Schulte, in *Solid Surf. Phys.* (Eds.: J. Hölzl, F.K. Schulte, H. Wagner), Springer Berlin Heidelberg, Berlin, Heidelberg, **1979**, pp. 1–150.
[5] G. N. Derry, M. E. Kern, E. H. Worth, *J. Vac. Sci. Technol. A Vacuum, Surfaces, Film.* **2015**, *33*, 060801.
[6] Y. Zhou, C. Fuentes-hernandez, J. Shim, J. Meyer, A. J. Giordano, H. Li, P. Winget, T. Papadopoulos, H. Cheun, J. Kim, M. Fenoll, A. Dindar, W. Haske, E. Najafabadi, T. M. Khan, H. Sojoudi, S. Barlow, S. Graham, J. Brédas, S. R. Marder, A. Kahn, B. Kippelen, Z. Yinhua, F.-H. Canek, S. Jaewon, M. Jens, G. A. J., L. Hong, W. Paul, P. Theodoros, C. Hyeunseok, K. Jungbae, F. Mathieu, D. Amir, H. Wojciech, N. Ehsan, K. T. M., S. Hossein, B. Stephen, G. Samuel, B. Jean-Luc, M. S. R., K. Antoine, K. Bernard, *Science (80-.).* **2012**, *336*, 327.
[7] S. P. Senanayak, B. Yang, T. H. Thomas, N. Giesbrecht, W. Huang, E. Gann, B. Nair, K. Goedel, S. Guha, X. Moya, C. R. McNeill, P. Docampo, A. Sadhanala, R. H. Friend, H. Sirringhaus, *Sci. Adv.* **2017**, *3*, e1601935.
[8] Z. Yinhua, F.-H. Canek, S. Jaewon, M. Jens, G. A. J., L. Hong, W. Paul, P. Theodoros, C. Hyeunseok, K. Jungbae, F. Mathieu, D. Amir, H. Wojciech, N. Ehsan, K. T. M., S. Hossein, B. Stephen, G. Samuel, B. Jean-Luc, M. S. R., K. Antoine, K. Bernard, *Science (80-.).* **2012**, *336*, 327.
[9] M. Kitamura, Y. Kuzumoto, W. Kang, S. Aomori, Y. Arakawa, *Appl. Phys. Lett.* **2010**, *97*, 33306.
[10] O. Fenwick, C. Van Dyck, K. Murugavel, D. Cornil, F. Reinders, S. Haar, M. Mayor, J. Cornil, P. Samorì, *J. Mater. Chem. C* **2015**, *3*, 3007.
[11] Y. Kuzumoto, M. Kitamura, *Appl. Phys. EXPRESS* **2014**, *7*, DOI 10.7567/APEX.7.035701.
[12] N. Fairley, V. Fernandez, M. Richard-Plouet, C. Guillot-Deudon, J. Walton, E. Smith, D. Flahaut, M. Greiner, M. Biesinger, S. Tougaard, D. Morgan, J. Baltrusaitis, *Appl. Surf. Sci. Adv.* **2021**, *5*, 100112.
[13] J. Leiro, E. Minni, E. Suoninen, *J. Phys. F Met. Phys.* **1983**, *13*, 215.
[14] C. Li, A. Guerrero, S. Huettner, J. Bisquert, *Nat. Commun.* **2018**, *9*, DOI 10.1038/s41467-018-07571-6.
[15] O. R. Yamilova, A. V. Danilov, M. Mangrulkar, Y. S. Fedotov, S. Y. Luchkin, S. D. Babenko, S. I. Bredikhin, S. M. Aldoshin, K. J. Stevenson, P. A. Troshin, *J. Phys. Chem. Lett.* **2020**, *11*, 221.
[16] G. Paul, S. Chatterjee, H. Bhunia, A. J. Pal, *J. Phys. Chem. C* **2018**, *122*, 20194.
[17] Q. Wang, Y. Shao, H. Xie, L. Lyu, X. Liu, Y. Gao, J. Huang, *Appl. Phys. Lett.* **2014**, *105*, DOI 10.1063/1.4899051.
[18] K. Liao, J. A. Yang, C. Li, T. Li, F. Hao, *ACS Appl. Mater. Interfaces* **2019**, *11*, 39882.
[19] B. Park, N. Kedem, M. Kulbak, D. Y. Lee, W. S. Yang, N. J. Jeon, J. Seo, G. Kim, K. J. Kim, T. J. Shin, G. Hodes, D. Cahen, S. Il Seok, *Nat. Commun.* **2018**, 1.
[20] V. Sharma, A. Herklotz, T. Z. Ward, F. A. Reboredo, *Sci. Rep.* **2017**, *7*, 1.
[21] Z. Ni, H. Jiao, C. Fei, H. Gu, S. Xu, Z. Yu, G. Yang, Y. Deng, Q. Jiang, Y. Liu, Y. Yan, J. Huang, *Nat. Energy* **2021**, *7*, DOI 10.1038/s41560-021-00949-9.
[22] Y. Xu, H. Sun, A. Liu, H. H. Zhu, W. Li, Y. F. Lin, Y. Y. Noh, *Adv. Mater.* **2018**, *30*, DOI 10.1002/adma.201801830.
